# A deformable *Bacillus subtilis* with gut homing for tumor treatment via bacterial metabolism-facilitated synergistic approach for starvation, chemotherapy, and immunotherapy

**DOI:** 10.1016/j.mtbio.2026.103295

**Published:** 2026-06-01

**Authors:** Tao Sun, Xiang Wang, Yawen Jiang, Mingxiu Liu, Lianting Huang, Limei Yang, Bei Guo, Kewei Wang, Guodong Sun, Yi Zhang, Wei Xue

**Affiliations:** a Guangdong Provincial Engineering and Technological Research Center for Drug Carrier Development, Department of Biomedical Engineering, Jinan University, Guangzhou, 510632, China; b College of Traditional Chinese Medicine, Jinan University, Guangzhou, 510632, China; c Guangdong Provincial Key Laboratory of Spine and Spinal Cord Reconstruction, the Fifth Affiliated Hospital (Heyuan Shenhe People's Hospital), Jinan University, Heyuan, 517000, China; d MOE Key Laboratory of Tumor Molecular Biology, Jinan University, Guangzhou, 510632, China; e College of Agricultural and Life Science, University of Wisconsin-Madison, Madison, WI 53706, USA

**Keywords:** Deformable *Bacillus subtilis*, Tumor microenvironment remodeling, Gut homing, Oncolytic bacterial therapy

## Abstract

Cancer treatment remains a critical global health challenge, which often requires multimodal and targeted therapeutic strategies, yet integrating these modalities typically demands complex formulations. Oncolytic bacterial therapy offers unique opportunities for targeted drug delivery and immune modulation, yet clinical translation has been hindered by safety concerns and tedious engineering modification. Here, we report a deformable *Bacillus subtilis* (DBS), constructed through one-step cisplatin-induced morphological transformation that elongates the bacterium while simultaneously incorporating chemotherapeutic payloads. The resulting DBS integrated three synergistic antitumor mechanisms: Chemotherapy, Starvation, and Immunotherapy. Metabolically, DBS actively consumes glucose and lactate within the tumor microenvironment (TME), thereby depriving tumor cells of key nutrients and reshaping the microenvironment to become more permissive to therapy. Concurrently, anaerobe-targeting DBS releases the encapsulated cisplatin locally in the TME, enabling direct cytotoxic killing while limiting systemic exposure. Furthermore, DBS leverages its intrinsic immunogenicity to potentiate immune cell activation, while its elongated morphology prolongs tumor retention and facilitates enhanced interactions with infiltrating immune cells. Proteases secreted by DBS hydrolyze the extracellular matrix, facilitating immune cell infiltration and boosting anti-tumor immune responses. Surprisingly, DBS exhibits intestinal homing and is safely cleared through fecal excretion after treatment, minimizing systemic side effects. This study presents an efficient and safe live-bacterial platform for cancer therapy, underscoring the broad potential of bacterial morphology engineering as a facile strategy to integrate multiple synergistic antitumor mechanisms within a single microbial therapeutic.

## Graphical abstract

**Figure:**
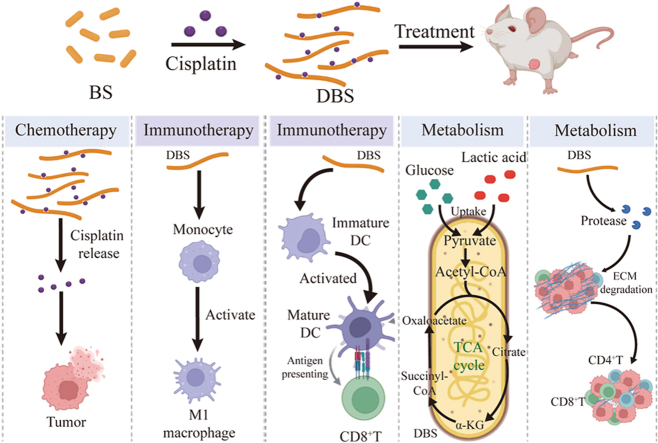

## Abbreviations

(BS): *Bacillus subtilis*
(DBS): Deformable *Bacillus subtilis*
(EC): *Escherichia coli*
(GFP): Green fluorescence protein
(TME): Tumor microenvironment

## 1 Introduction

**Scheme 1:**
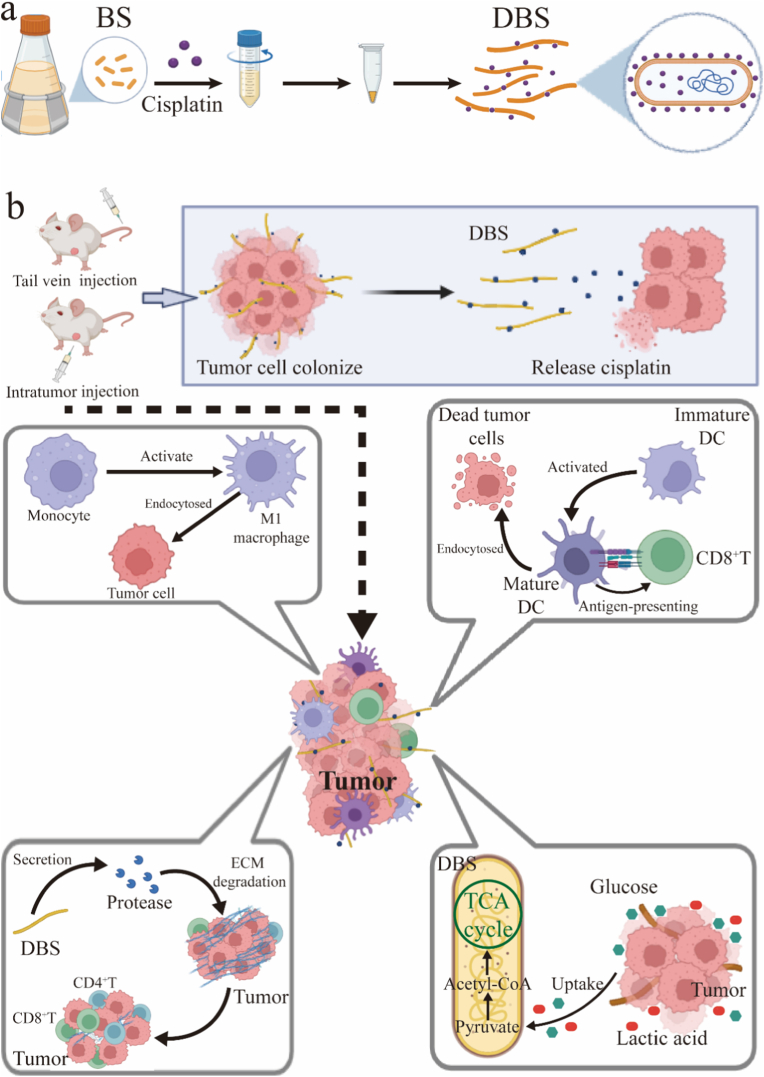
**Schematic illustration of DBS preparation, administration, and proposed antitumor mechanisms**. a) Preparation process of DBS: Probiotic BS was incubated with cisplatin to induce filamentous morphological changes, yielding cisplatin-laden DBS exhibiting elongated shape. b) Proposed mechanisms of DBS-mediated antitumor effects following intratumoral injection or tail vein injection: 1) DBS directly kill tumor cells by releasing cisplatin (top). 2) DBS stimulate M1 macrophage polarization, enhancing innate immunity (middle left), and promote dendritic cell maturation and CD8^+^ T cell activation (middle right). 3) Tumor stroma remodeling by DBS-secreted proteases enhances anti-tumor immune cell infiltration (bottom left). 4) DBS mediates anti-tumor effects through glucose consumption, inducing tumor starvation, while lactate metabolism contributes to TME remodeling. (bottom right).

Cancer remains one of the leading causes of mortality worldwide [1,2]. Chemotherapy, while a cornerstone of cancer treatment, frequently proves insufficient for effective tumor control due to its inherent limitations such as drug resistance and the resulting systemic toxicities. Therefore, the strategic coordination of multiple treatment modalities is the prevailing approach in current clinical practice. Numerous studies suggest that introducing exogenous microorganisms such as bacteria into tumors can achieve meaningful therapeutic outcomes [3,4]. Unlike traditional treatments, bacterial cancer therapy leverages living microorganisms to stimulate antitumor immunity while simultaneously functioning as active metabolic modulators. Owing to the aberrant metabolic features of tumor tissues, such as elevated energy dependence and an acidic microenvironment, these characteristics can be exploited as therapeutic targets by metabolically active bacteria. Consequently, metabolically capable bacteria can leverage these vulnerabilities as therapeutic targets. In addition, solid tumors typically exhibit low-oxygen and nutrient-rich microenvironment, presenting an ideal niche for colonization of anaerobic bacteria. Nevertheless, the efficacy of bacterial cancer therapy is often compromised by the rapid clearance of bacteria from tumors, which greatly diminishes their antitumor efficacy, thereby establishing prolonged bacterial retention as a key requirement for achieving effective tumor treatment [5,6]. Current strategies often employ surface coatings of bacteria to increase their retention time in tumors, while being useful, it frequently impairs the intrinsic activity and function of the encapsulated bacteria [7]. In contrast, modulating the morphological characteristics of bacteria could provide a simple yet highly effective way to enhance bacterial retention within tumors. So far, research on modulating bacterial morphology to enhance cancer treatment remains largely unexplored. However, accumulating evidence suggests that bacterial morphological characteristics may significantly influence their interactions with host tissues, organs, and the immune system, ultimately impacting their therapeutic efficacy against tumors [8,9] (see Scheme 1).

Bacteria can adapt to external environmental stresses by transitioning to filamentous forms. For instance, *Pseudomonas aeruginosa* elongates under high shear stress in fluid dynamics, while *Escherichia coli* (EC) exhibits filamentation in response to sub-lethal heat or cold, offering a basis for morphology manipulation [10]. These morphological changes, characterized by increased length, surface area, and aspect ratio, occur without altering the bacterial species identity and can markedly influence the interactions between the bacteria and the host [11,12]. Notably, research has revealed that bacterial morphological changes can be induced not only by physical cues, but also through treatment with chemical substance. We highlight that cisplatin, a widely used chemotherapeutic agent for diverse cancers, is capable of inducing substantial morphological transformation in bacteria, and simultaneously, being effectively loaded within the bacterial cells. This represents a novel and microbial-driven mode of drug loading, where the therapeutic agent actively contributes to both bacterial engineering and its integrated packaging and delivery [13].

*Bacillus subtilis* (BS), a well-characterized probiotic, offers various advantages for cancer therapy due to its hypoxia tropism, potential as a bio-carrier for drug delivery, and potent immunomodulatory capabilities [14,15]. Emerging evidence highlights the excellent safety profile of BS, paving the way for its administration without significant adverse effects [16]. Previously, BS has shown promise in maintaining intestinal health and treating gastrointestinal disorders owing to its robust biological activity and versatility. The anti-tumor potential of BS largely stems from its unique cell wall components, including exposed polysaccharides and proteins that can directly interact with host immune cells and trigger potent anti-tumor immune responses. Furthermore, BS possesses robust metabolic capabilities, particularly the ability to consume key nutrients such as glucose, which are vital for tumor survival. This metabolic competition not only contributes to its anti-tumor activity but also provides a foundation for synergistic effects when combined with multimodal therapeutic strategies [17]. Inspired by this, we aim to investigate how cisplatin-induced morphological changes in BS impact its antitumor efficacy, integrating cisplatin's chemotherapeutic action with BS's innate advantages, including metabolic activities and immunogenicity.

**Fig. 1:**
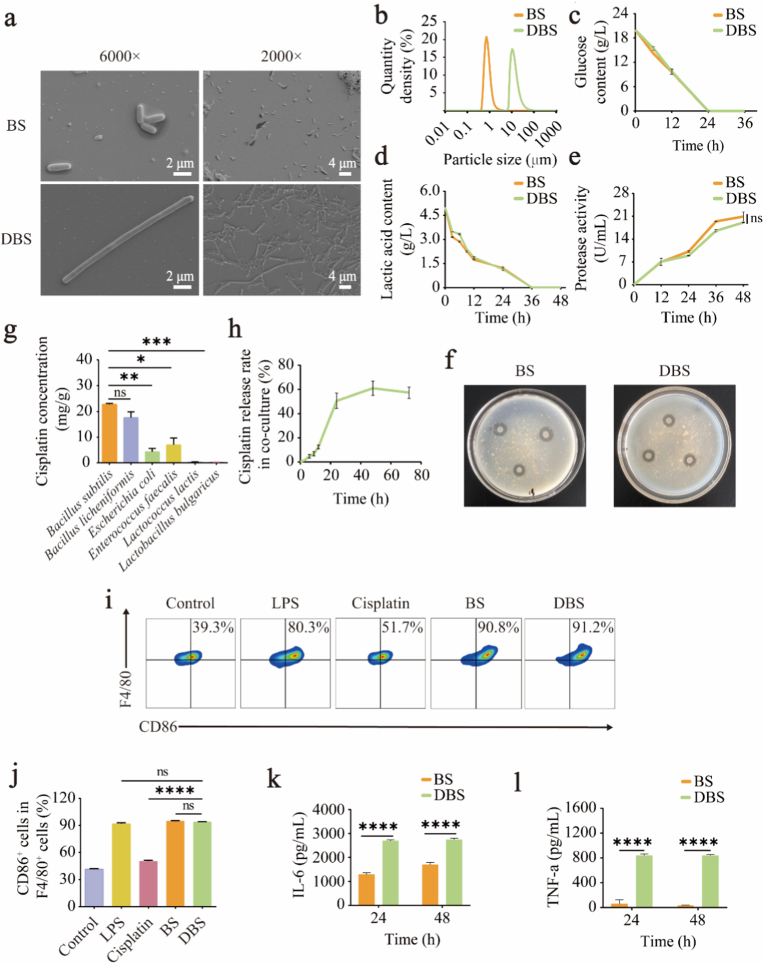
**Morphology, Metabolism, Drug Release, and In Vitro Immune Response of BS and DBS.** a) SEM images of BS and DBS mycelium (Scale bar: 2 μm or 4 μm). b) Quantitative size analysis of bacterial cells. c) Glucose concentration in bacterial cultures over time. d) Lactic acid concentration in bacterial cultures over time. e) Evaluation of protease activity in bacterial cultures over time. f) Images of hydrolysis zones formed by BS and DBS-secreted proteases on milk agar plates. g) Quantification of cisplatin loading in bacterial pellets of various strains, presented as mg of cisplatin per g of bacterial pellets. h) Cisplatin release profile of DBS co-cultured with 4T1 cells. i) Representative flow cytometry analysis of M1 macrophages (F4/80^+^CD86^+^) and quantitative analysis. j) Cytokine secretion levels of macrophages co-cultured with BS or DBS: k) IL-6, l) TNF-α. All quantified results are expressed as mean ± SD (*n* = 3, biological triplicate).

In this study, we engineered Deformable *Bacillus subtilis* (DBS) by facile inducing filamentous morphological changes of BS using cisplatin, which acted both as a morphological modulator and a chemotherapeutic payload synchronously (Fig. 1). This design aimed to create a live bacterial platform capable of tumor targeting, sustained immune activation, metabolic remodeling of the TME and localized drug release. In mouse tumor models, DBS demonstrated multiple advantages. At a systemic level, it effectively prolonged intratumoral retention benefit from elongated morphology, facilitating sustained activation of immune cells to eliminate tumor cells. At the micro level, utilizing its metabolic capability, DBS can deplete glucose within tumor tissues, inducing a state of metabolic starvation that directly inhibits tumor cell proliferation. Furthermore, the ability of DBS to remodel the TME is mediated by two key processes: the consumption of tumor-promoting immunosuppressive factors (e.g., lactic acid) through its metabolism, and the degradation of tumor interstitial matrix via secreted proteases. Consequently, DBS alleviated the immunosuppressive TME and enhanced the penetration of cisplatin and immune cells, synergistically boosting the host's antitumor immunity and chemotherapy response. The development of effective and highly safe microbial cancer therapeutics remains a long-lasting challenge in this field. For the first time, we discovered that DBS are prone to translocation and clearance through the gastrointestinal tract following therapeutic task. This clearance is instrumental in mitigating the risk of prolonged systemic exposure and associated toxicities, distinguishing it from conventional bacterial vectors that tend to persist in the body. Concurrently, the presence of DBS in the Gastrointestinal (GI) tract is associated with beneficial modulation of the gut microbiota composition, contributing to an improved overall host health profile. In contrast to its original form BS, DBS demonstrates a significantly enhanced capacity to activate both innate and adaptive immune responses, thereby potently suppressing tumor growth. Crucially, when benchmarked against commonly employed bacterial therapeutics, such as EC, DBS emerges as a demonstrably safer and more reliable platform strain for tumor immunotherapy. This study highlights the potential of engineering bacterial morphology as a simple yet powerful strategy for enhancing microbial cancer therapy, potentially broadening its translation potential.

## 2 Results and discussion

### 2.1 Induction and characterization of DBS

Previous studies have shown that certain bacterial strains undergo filamentous morphological changes upon exposure to cisplatin [18,19]. Cisplatin induces DNA damage in *Bacillus subtilis* (BS), leading to filamentation, a phenomenon where cells continue to grow without dividing, ultimately resulting in longitudinal elongation and the formation of filamentous structures, and this interference with bacterial division occurs without directly killing the bacteria [20,21]. We first screened the morphological deformation of commonly used model bacterial strains (*Bacillus subtilis* strain 168 (wild type), *Bacillus licheniformis* ATCC 14580, *Enterococcus faecalis* OG1RF, *Escherichia coli* BL21 (DE3), *Pediococcus acidilactici* DSM 20284, and *Lactobacillus bulgaricus* ATCC 11842) in the presence of cisplatin (Fig. S1). Among them, BS exhibited the most notable cisplatin-induced filamentation, with an aspect ratio reaching 20:1, making it a suitable model for investigating bacterial deformation and its effect on tumor treatments. To confirm the morphological changes of BS before and after cisplatin treatment, scanning electron microscopy (SEM) was employed to reveal distinct morphological differences. Under untreated conditions, BS cells averaged approximately 2 μm in length. Following cisplatin treatment, however, the cells underwent dramatic elongation, reaching an average length of about 20 μm, a nearly 10-fold increase. This remarkable increase in length significantly amplified the cells' aspect ratio, while their width remained largely unchanged (Fig. 1a and b). These results demonstrated that cisplatin treatment induced a marked morphological transformation in BS, successfully establishing DBS.

Subsequently, we evaluated whether the cisplatin-induced filamentation of BS is reversible. Cisplatin-treated BS cells were washed and resuspended in fresh LB medium without drug. Samples taken at 0, 6, 12, 24 and 48 h showed a gradual shortening of the elongated cells, and by 48 h the mean cell length was indistinguishable from that of untreated controls, indicating that the morphological deformation is fully reversible (Fig. S3). To test the specificity of this response, DBS was also exposed to 120 ng/mL of 5-fluorouracil (5-FU), doxorubicin (DOX) or paclitaxel (PTX). Both 5-FU and PTX caused modest elongation, whereas DOX produced only a slight, transient increase. All three agents elicited far less pronounced filamentation than cisplatin (Fig. S4), demonstrating that filamentation is not exclusive to cisplatin but represents a general bacterial stress response to diverse DNA-damaging or cytotoxic drugs. Second, we evaluated whether DBS retained the viability and metabolic characteristics of BS, in view of its intended application. DBS grew robustly under different pH conditions as the BS, reaching an average OD_600_ above 6.5 after 48 h, suggesting their biological activity remained unimpaired (Fig. S5). Plate-counting of colony-forming units showed no statistically significant difference between DBS and BS, thereby confirming that the cisplatin-loaded, filamentous bacteria retain biological activity comparable to that of untreated *B. subtilis* (Fig. S6). Using high-performance liquid chromatography (HPLC), we assessed DBS's metabolic capacity for glucose and lactate. DBS consumed glucose (Fig. 1c) and metabolized lactate in a similar manner to BS within 36 h (Fig. 1d), indicating its metabolic capabilities were maintained. In addition, protease activity assays revealed a time-dependent increase in the total protease activity in the DBS culture, indicating that DBS bacteria progressively secrete and accumulate proteases over time (Fig. 1e). This was further supported by the presence of clear hydrolysis zones around DBS colonies on milk agar plates (Fig. 1f), indicative of protease activity comparable to that of BS. We also assessed DBS's ability to utilize glutamine. DBS exhibited robust growth and efficiently consumed glutamine when cultured in glutamine-containing medium (Fig. S7), further demonstrating its metabolic versatility. Furthermore, we utilized Inductively Coupled Plasma Mass Spectrometry (ICP-MS) to determine the platinum (Pt) content loading in DBS. Interestingly, in addition to the most pronounced length increase compared to other bacterial strains, DBS exhibited the highest cisplatin loading capacity (Fig. 1g and Fig. S2). A direct correlation was observed between the morphological elongation of DBS and its enhanced drug loading capacity. The increased aspect ratio enlarges the available surface area, thereby improving cisplatin adsorption and uptake and resulting in higher intracellular Pt content. Therefore, the remarkable length increase in DBS under cisplatin treatment is not merely a morphological artifact but likely a key indicator of enhanced drug uptake and retention. This synergistic interplay between deformation extent and increased drug affinity positions DBS as a promising candidate for further investigation as a potential probiotic-based drug delivery vehicle. The ability of DBS to effectively load high levels of platinum compounds underscores its potential utility in developing targeted anticancer therapeutic strategies.

DBS was generated using the anticancer drug cisplatin, leading to its incorporation within the bacterial structure. We therefore investigated the ability of DBS to release cisplatin, thereby functioning as a living drug delivery system. The drug release profile of cisplatin from DBS was investigated using a co-culture with 4T1 breast cancer cells, mimicking the tumor microenvironment (TME), where 60% of loaded cisplatin was continuously released from DBS in around 48 h (Fig. 1h). Moreover, co-culture experiments showed that DBS treatment caused a marked decrease in the number of viable 4T1 cells relative to the BS or untreated control groups (Fig. S11). The same inhibitory effect was observed when DBS was co-cultured with the B16 melanoma cell line and the HCT-8 human colorectal-cancer cell line (Fig. S12) [[22], [23], [24]]. These findings demonstrate the robust cytotoxic activity of cisplatin released from DBS, leading to enhanced killing of 4T1 cancer cells. Concurrently, DBS competed with 4T1 cells for nutrients such as glucose, further contributing to 4T1 cell death.

Collectively, these findings demonstrate that cisplatin treatment induces not only filamentation in BS but also a concurrent loading of the chemotherapeutic agent. Crucially, this morphologically altered BS retains its original viability and metabolic/secretory functions, while enabling sustained release of incorporated cisplatin. This highlights the potential of DBS as a natural living carrier for the anticancer drug cisplatin, making it highly suitable for our subsequent antitumor applications.

### 2.2 Macrophage activation and cytokine secretion *in vitro*

Previous research indicated bacteria are able to induce macrophage polarization and elicit robust antitumor immune responses [25,26]. Therefore, we next sought to investigate whether DBS also exhibited these immunostimulatory effects. As an initial *in vitro* assessment of its immunomodulatory potential, RAW264.7 macrophages were co-cultured with DBS cells to assess its immunostimulatory potential, in comparison to LPS (a well-established immunostimulant), pure cisplatin, BS, and a negative control (PBS) (Fig. 1i). Compared to the PBS control, treatment with LPS, BS, and DBS significantly increased the proportion of M1 macrophages (Fig. 1j). While pure cisplatin also demonstrated a modest induction of M1 polarization, its effect was similar to the negative control. This finding strongly suggests that the observed pro-M1 polarization effect of BS and DBS is primarily attributed to their bacterial components, rather than the presence of chemotherapy drugs. Notably, no significant differences were observed between BS and DBS in M1 polarization when compared to LPS, indicating that surface components such as lipoteichoic acid, peptidoglycan fragments, or lipoproteins present on both BS and DBS contributed to macrophage polarization, and that DBS retained the immunostimulatory properties of the original strain [27].

Various studies have shown that the morphology of pathogen can influence their immunogenicity [28,29]. To investigate whether the elongated structure of DBS affected its immunostimulatory properties, we measured cytokine secretion. Co-culture of RAW264.7 macrophages with BS or DBS resulted in significant increases in the levels of IL-6 (Fig. 1k) and TNF-α (Fig. 1l), demonstrating that DBS retained its immunostimulatory capacity to stimulate the immune system. Cytokine release in response to DBS was significantly higher than that induced by BS, suggesting that the morphological alteration not only preserved immunogenicity but also enhanced immune activation. To probe the role of morphological plasticity in DBS-mediated phagocytosis by macrophages, we employed FITC labeling of both BS and DBS followed by co-incubation. Phagocytosis assays revealed distinct kinetics: uptake of BS peaked rapidly and plateaued within 3 h, whereas phagocytosis of DBS continued progressively, leading to a significantly higher level at 24 h. This indicates that the morphological change to DBS delays or prolongs the phagocytosis process, resulting in a slower but sustained uptake by macrophages over time (Fig. S13). Consequently, DBS-containing macrophages might persist for a longer duration. When administered *in vivo*, this characteristic could prolong its retention time, thus stimulating sustained immune activation. The observed delayed phagocytosis suggests an extended extracellular contact time between DBS and macrophages. This prolonged interaction could lead to continuous stimulation of macrophage surface receptors, including Toll-like receptors 2 (TLR2) and 4 (TLR4) [30]. Consequently, this sustained receptor engagement may drive an increase in pro-inflammatory factor production. This scenario directly supports our hypothesis that the altered morphology of DBS significantly augments its immunogenicity. Therefore, if utilized as an *in vivo* therapeutic agent, the extended morphology of DBS could potentially prolong its retention time, thereby sustaining immune stimulatory effect over an extended period.

Our results demonstrate that DBS, an engineered BS strain with cisplatin-induced filamentous morphology, elicits enhanced immunomodulatory effects compared to unmodified BS. Collectively, these findings highlight the immunotherapy potential of DBS as a living anticancer agent.

### 2.3 Enhanced tumor accumulation and prolonged retention of DBS

**Fig. 2:**
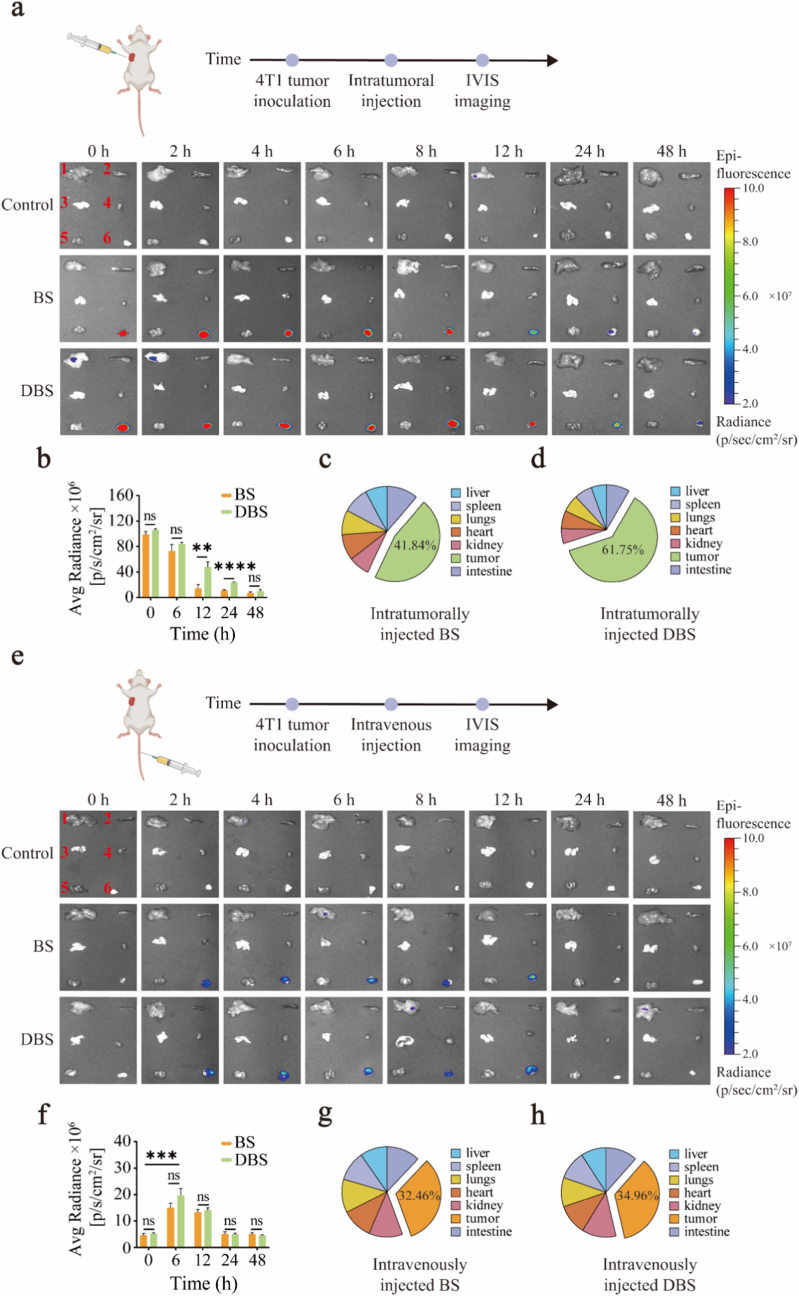
**Tumor targeting and retention of BS-GFP and DBS-GFP in 4T1 tumor-bearing mice following tail vein or intratumoral injection.** a) Schematic of experimental design for intratumoral injection and representative fluorescence images of major organs (1. liver, 2. spleen, 3. lung, 4. heart, 5. kidney) and tumor (6) after intratumoral injection. b) Quantitative analysis of tumor fluorescence over time post-intratumoral injection. c-d) Fluorescence distribution in organs and tumor after intratumoral injection at 12 h: c) BS. d) DBS. e) Schematic of experimental design for tail vein injection and representative fluorescence images of major organs (1. liver, 2. spleen, 3. lung, 4. heart, 5. kidney) and tumor (6) after tail vein injection. f) Quantitative analysis of tumor fluorescence over time post-tail vein injection. g-h) Fluorescence distribution in organs and tumor after tail vein injection at 12 h: g) BS. h) DBS. All quantified results are expressed as mean ± SD (n = 5, biological replicate).

Building upon our findings that DBS exhibited phagocytosis resistance *in vitro*, we next investigated their *in vivo* fate, specifically focusing on their distribution profile within tumor and main organs. To visualize bacteria, we genetically engineered BS to express green fluorescent protein (GFP) (Fig. S14). Successful transformation of BS was confirmed by DNA electrophoresis (Fig. S15 and S16) and fluorescent imaging (Fig. S17). A 4T1 breast cancer model was established in mice subcutaneously, followed by intratumoral administration of BS-GFP or DBS-GFP. Real-time fluorescence in tumor and organs was monitored using an *in vivo* imaging system (IVIS) (Fig. 2a). Fluorescence imaging over time demonstrated strong fluorescence signals of both but more sustained signals of DBS-GFP in tumors compared to BS-GFP (Fig. 2b). Over a longer observation period (12-48 h), DBS-GFP maintained significantly higher fluorescence intensity compared to BS-GFP, whose signals diminished more rapidly (Fig. S18). The enhanced persistence of DBS in tumor represents a significant advancement over conventional microbial therapies. While previous studies reported that engineered EC has a retention time of 12 h in tumors [31], DBS exhibited notably prolonged retention, with detectable fluorescence signals still observable at the tumor site 48 h post-administration. Moreover, our *in vivo* biodistribution studies at 24 h post-administration revealed a significantly higher overall accumulation of engineered DBS compared to the original BS strain in tumor (61.75% for DBS vs. 41.84% for BS; Fig. 2c and d). The extended retention is likely because DBS have enhanced resistance to immune clearance such as phagocytosis by macrophage, as we observed *in vitro*. This extended retention suggests DBS as a potential platform for sustained drug release and immune stimulation within the tumor.

Given that our intratumoral administration is not fully relevant to clinical applications, and BS is a facultative anaerobic bacterium with a tendency to target hypoxic environments [32]. Consequently, we proceeded to the systemic administration of BS-GFP and DBS-GFP via tail vein injection. IVIS revealed that both BS-GFP and DBS-GFP exhibited detectable fluorescent signals in the tumor as early as 2 h post-injection, peaking at 6 h before gradually declining (Fig. 2e and f) as shown in long-term monitoring of BS-GFP and DBS-GFP within tumor tissues (Fig. S19) and quantified fluorescence distribution across tumor and major organs at 12 h (Fig. 2g and h). The majority of fluorescence for both BS and DBS was localized to tumors, and no significant differences in organ distribution were observed between BS and DBS. These results indicate that DBS retains tumor-targeting capabilities comparable to BS, and its elongated morphology does not impair targeting efficiency. We quantified the platinum content in the major organs 6 h after administration of either free cisplatin or cisplatin-loaded *Bacillus subtilis* (DBS) via two routes: tail-vein injection and direct intratumoral injection. In both delivery modalities, DBS produced a significant reduction in cisplatin accumulation in non-target (off-target) organs compared with the free drug (Fig. S20). This diminished off-target distribution indicates that DBS markedly lowers systemic exposure and, consequently, the collateral side effects associated with conventional cisplatin therapy.

Collectively, these findings demonstrate that DBS exhibits tumor-targeting retention capabilities regardless of the administration route. Crucially, the elongated morphology of DBS does not impede its tumor-targeting efficacy via systemic circulation, suggesting DBS as a promising candidate for targeted cancer therapy.

### 2.4 Antitumor effects through intratumoral injection of DBS

**Fig. 3:**
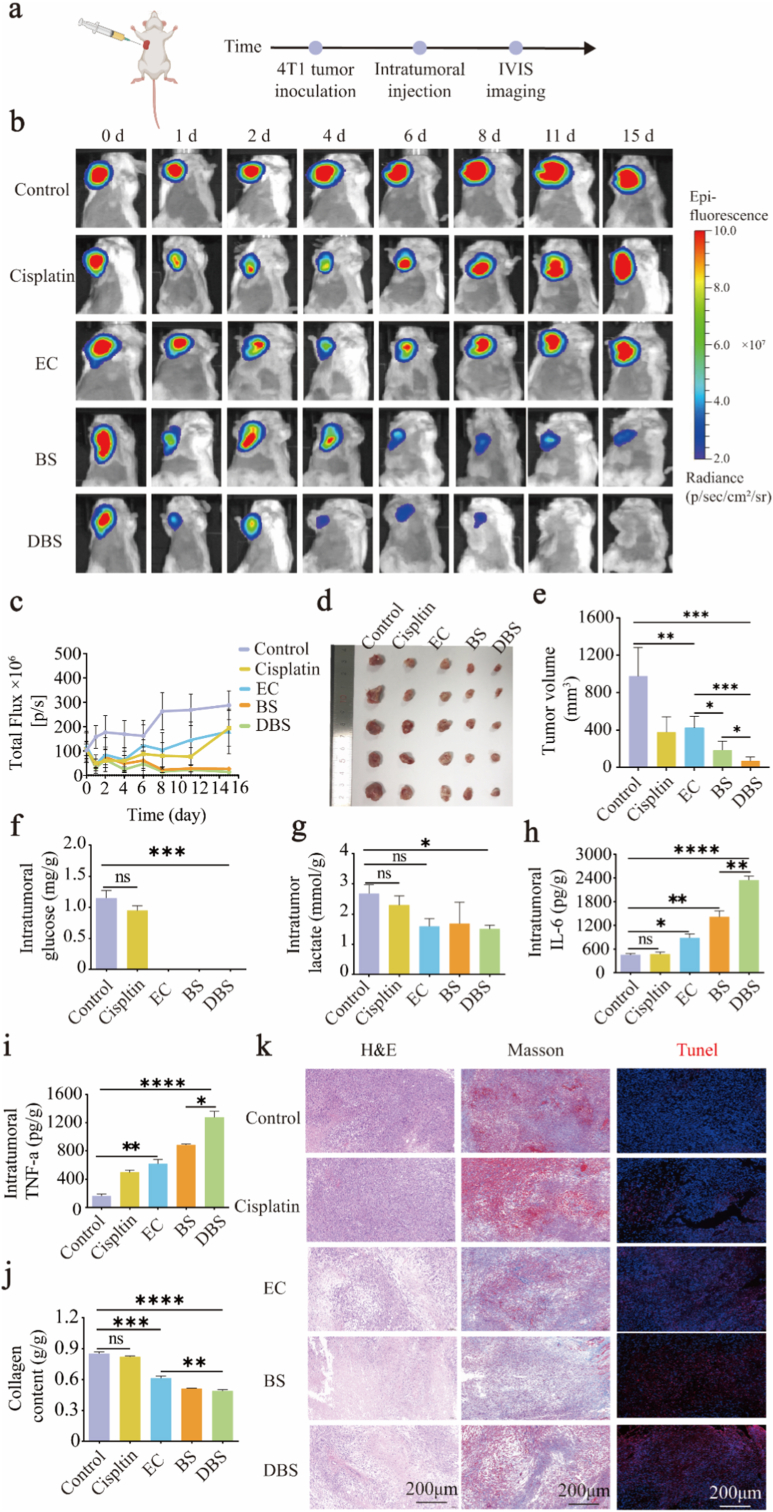
***In vivo* antitumor efficacy, safety evaluation, and TME modulation of BS and DBS following intratumoral injection.** a) Schematic diagram of the tumor treatment protocol via intratumoral injection. b) Representative bioluminescence images of tumor-bearing mice after intratumoral injection of various therapeutic regimens. c) Quantification of bioluminescence signals over time. Data are presented as mean ± SD, *n* = 5. d) Image of excised tumors after intratumoral therapy. e) Quantification of tumor volume after therapy, presented as mean ± SD (n = 5). f) Intratumoral glucose levels after intratumoral therapy (mean ± SD, *n* = 3). g) Intratumoral lactate levels after intratumoral therapy (mean ± SD, *n* = 3). h) Intratumoral IL-6 levels after intratumoral therapy (mean ± SD, *n* = 3). i) Intratumoral TNF-α levels after intratumoral therapy (mean ± SD, *n* = 3). j) Quantitative analysis of collagen content in tumor tissues after intratumoral therapy (mean ± SD, *n* = 3). k) Tissue samples examined for histological morphology through H&E, Masson staining and apoptosis evaluation via TUNEL staining (TUNEL displays red fluorescence and nuclei are stained in blue, scale bar: 200 μm).

Based on DBS's tumor targeting and retention abilities, we evaluated its antitumor effects by intratumoral injection (Fig. 3a). Intratumoral injection can achieve high bacterial concentrations directly at the tumor site, which is helpful to maximize DBS's antitumor effects as the first trial. The results demonstrated that both BS and DBS treatment elicited significantly more pronounced antitumor effects than EC or pure cisplatin (Fig. 3b and c). This superiority is likely attributed to the stronger antitumor immune responses elicited by BS and its greater ability to degrade extracellular matrix components compared with EC, as confirmed by our subsequent results. DBS further amplified these advantages through cisplatin loading and its elongated morphology, leading to the most potent therapeutic outcome (Fig. 3d and e).

Reshaping the TME is a critical strategy in cancer immunotherapy, as it enhances therapeutic responsiveness, alleviates immunosuppression, and ultimately improves therapeutic outcomes and prognosis [33]. Given our above *in vitro* validation on the robust glucose and lactate metabolism of BS and DBS, we analyzed intratumoral glucose and lactate concentrations. Compared to control groups, both BS and DBS treatments led to a significant reduction in intratumoral glucose and lactate levels (Fig. 3f and g). This depletion of glucose by BS and DBS effectively induced nutritional starvation in tumor cells, thereby exerting a direct cytotoxic effect. Concurrently, the decrease in lactate, a key immunosuppressive metabolite, directly reversed the immunosuppressive milieu within the TME. These findings collectively indicate that BS and DBS treatment remodeled the TME into a state unfavorable for tumor growth and progression. Furthermore, DBS treatment elicited the most potent induction of intratumoral IL-6 and TNF-α among all groups, significantly surpassing that of BS and EC (Fig. 3h and i). This observation strongly suggests that DBS promoted superior local immune activation. A potential mechanism for this enhanced immunogenicity may lie in the prolonged exposure of bacterial Pathogen-Associated Molecular Patterns (PAMPs) due to the elongated shape and the extended retention time of DBS.

To further investigate the mechanisms underlying the superior antitumor efficacy of DBS, we performed histological analyses on resected tumors to visually investigate the role of DBS in inhibiting tumor growth. As shown in Fig. 3k, Hematoxylin and Eosin (H&E) staining of resected tumor tissues revealed a significantly increased area of necrotic tumor cell nuclei in the DBS treatment group, while terminal deoxynucleotidyl transferase dUTP nick end labeling (TUNEL) staining showed the highest fluorescence intensity, confirming robust tumor-killing. The quantitative TUNEL results showed that the DBS group had the highest percentage of apoptotic cells (Fig. S27). Tumor cells often promote excessive deposition of extracellular matrix (ECM) components, such as collagen and hyaluronic acid, which increases tissue stiffness and impedes interstitial fluid flow, posing a significant physical barrier to drug and immune cell penetration into tumors. Notably, Masson staining showed the collagen content had significantly decreased in the DBS group, suggesting that DBS reduced collagen proteins through their proteolytic activity, thereby significantly ameliorating interstitial fluid pressure (IFP). Furthermore, quantitative collagen measurements (Fig. 3j) further confirmed a significant reduction in collagen content, with DBS showing the most pronounced decrease.

Throughout the treatment period, no significant weight loss was observed, indicating that intratumoral administration of bacteria did not introduce substantial systemic side effects (Fig. S21). We then evaluated the systemic inflammation by measuring serum level of several biomarkers. No significant increase of inflammation-associated biomarkers C-reactive protein (CRP) and procalcitonin (PCT) levels were observed in BS and DBS groups (Figs. S23 and S24). Serum level of proinflammatory cytokines IL-6 and TNF-α (Fig. S25 and S26) were slightly elevated in the BS and DBS groups, but significantly lower than EC-treated groups, indicating favorable safety of BS and DBS than EC (widely used engineering bacteria). Moreover, liver and kidney function parameters remained within normal ranges following treatment with DBS. In addition, histological analyses of major visceral organs by H&E staining further showed no detectable tissue damage or inflammation (Fig. S32). These findings collectively suggest a considerable safety profile for DBS treatments.

In conclusion, these findings collectively demonstrate that DBS functions as a potent bacterial therapeutic capable of exerting significant antitumor effects. Its efficacy is further enhanced by the combined actions of cisplatin release, activation of antitumor immunity, and remodeling of TME. Together, these mechanisms form a coordinated therapeutic strategy that integrates chemotherapy, immunotherapy, and metabolic starvation, resulting in a robust and multifaceted anticancer response.

### 2.5 Activation of antitumor immunity by DBS

**Fig. 4:**
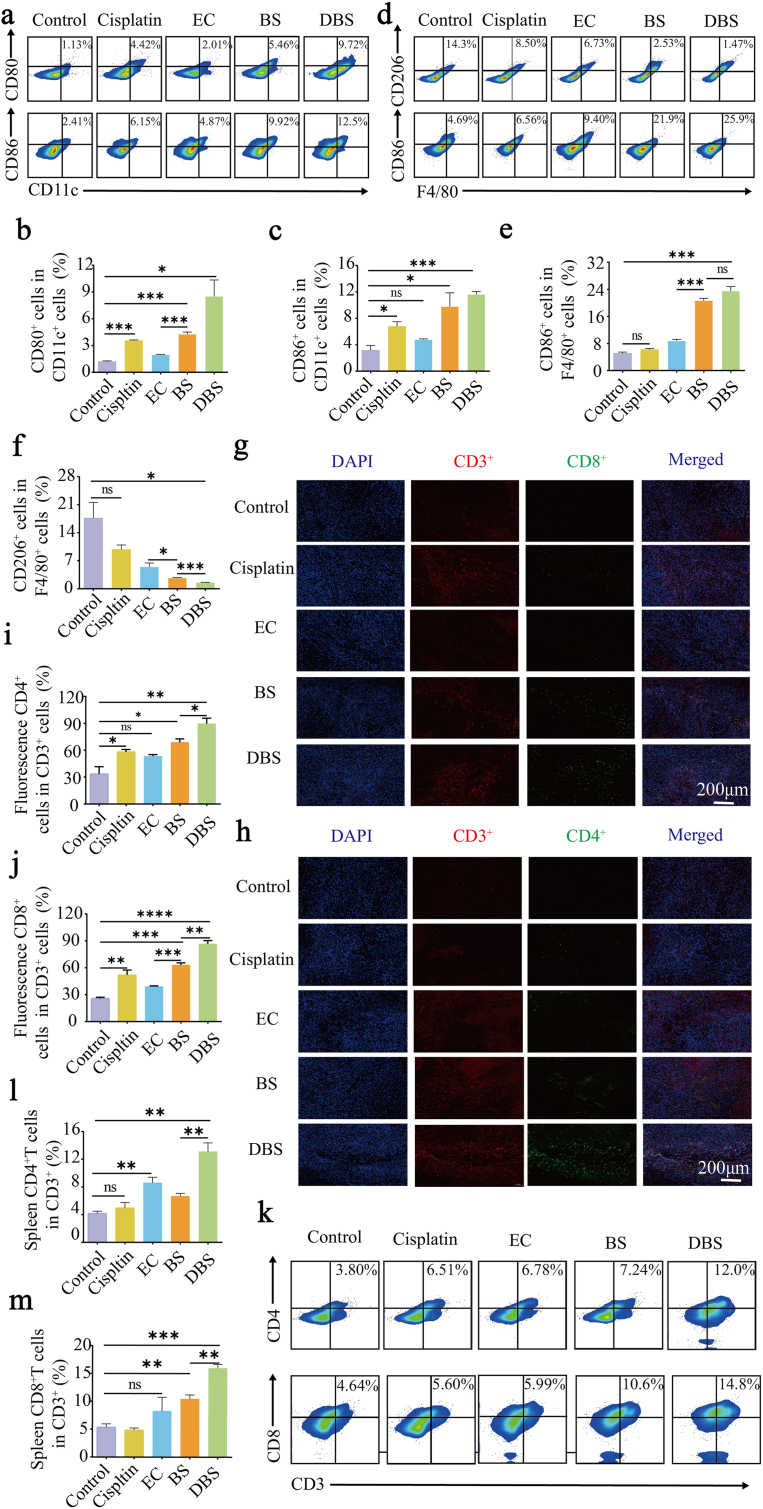
**Immune activation analysis in tumor-bearing mice.** a) Representative flow cytometry analysis of matured dendritic cells (CD80^+^ and CD86^+^ cells) proportions in CD11c^+^ cells in peri-tumoral lymph nodes after intratumoral injection and the quantitative percentage of CD80^+^ cells b) and CD86^+^ cells c). d) Representative flow cytometry analysis of M1 (CD86^+^) and M2 (CD206^+^) phenotype proportions in macrophage (F4/80^+^) in tumor tissue after intratumoral therapy and the quantitative percentage of CD86^+^ M1 macrophage e) and CD206^+^ M2 macrophage f). g) h) Immunofluorescence images of CD4^+^ and CD8^+^ T cells (CD3^+^) in tumor sections after intratumoral therapy. CD3^+^ markers are stained in red, whereas CD4^+^ or CD8^+^ markers are stained in green. Nuclei are stained in blue using DAPI. Scale bar: 200 μm. Quantification of CD4^+^ and CD8^+^ T cell infiltration within CD3^+^ T cell populations of tumor sections post-intratumoral treatment. ImageJ was used to determine the percentage of CD4^+^ T cells (i) and CD8^+^ T cells (j) within the CD3^+^ T cell gate. T cell infiltration in spleen after different treatments, representative flow cytometric analysis of CD4^+^CD8^+^ T cells (CD3^+^CD4^+^CD8^+^) k) and quantification (l) (m), with quantitative data expressed as mean ± SD (n = 3). All quantified results are expressed as mean ± SD (n = 3, biological triplicate).

As we demonstrated the potent antitumor efficacy of DBS, and given DBS's potential to elicit stronger immune response intratumorally, we next investigated its capacity to stimulate antitumor immunity. Dendritic cells (DCs) are pivotal in initiating antitumor immunity by capturing and presenting tumor-associated antigens to T cells, thereby bridging innate and adaptive immune responses [34]. Following three intratumoral injections of therapeutics to tumor-bearing mice, we first harvested the peri-tumoral lymph nodes near the tumor site and analyzed the proportion of mature DCs using flow cytometry (Fig. 4a). Intratumoral treatment with DBS and BS significantly increased the percentage of mature DCs populations in peri-tumoral lymph nodes compared to other groups, including the EC-positive control (Fig. 4b and c). Notably, the DBS treatment group exhibited the highest level of DCs maturation. This enhanced maturation suggests that the morphological elongation and enhanced retention of DBS promote increased interaction with host immune cells, leading to augmented antigen presentation. Although EC also induced DCs activation, LPS from EC is a potent TLR4 agonist, and it is readily shed and rapidly enters the circulation, where it drives systemic inflammation (Figs. S25 and S26) [35]. In contrast, DBS presents Gram-positive PAMPs such as peptidoglycan and lipoteichoic acids, which are more stable and efficiently recognized by TLR2 expressed on DCs and macrophages in the tumor and draining lymph nodes [36]. This effectively promotes DCs activation and further immune response within the TME.

Macrophages, as central players in innate immunity, significantly influence the TME through their polarization states [37]. Within a tumor-suppressive TME, macrophages predominantly adopt an anti-inflammatory M2 phenotype. Conversely, M1-polarized macrophages possess potent anti-tumor capabilities, including the release of pro-inflammatory cytokines, the production of cytotoxic mediators such as reactive oxygen species (ROS) and nitric oxide (NO), and enhanced antigen presentation [38]. We analyzed the proportions of M1 and M2 macrophages within the harvested tumor tissues using flow cytometry (Fig. 4d). While both BS and DBS treatment groups induced a significant increase in the proportion of M1 macrophages (CD86^+^ gated on F4/80^+^) and a corresponding decrease in the M2 population (CD206^+^ gated on F4/80^+^), there was a notable trend suggesting that DBS treatment induced a stronger ratio of M1 to M2 macrophages compared to BS (Fig. 4e and f). This finding aligns with the *in vitro* macrophage stimulation results, indicating that DBS preserves and enhances the immunogenicity of BS that effectively promotes macrophage polarization toward a proinflammatory, antitumor phenotype.

Based on the observed activation of innate immunity, we next examined the adaptive immune responses, focusing on tumor-infiltrating T cells. CD8^+^ cytotoxic T cells serve as direct effectors in eliminating malignant cells, while CD4^+^ helper T cells sustain cytotoxic function and coordinate diverse immune mechanisms [39]. To further validate whether DBS treatment elicits a more effective anti-tumor adaptive immune response, we performed immunofluorescence (IF) staining on excised tumor tissues (Fig. 4g and h) and subsequently quantified the fluorescence intensity (Fig. 4i and j). Our results revealed increased accumulation of CD4^+^ and CD8^+^ fluorescence signals in CD3^+^ T cells specifically within DBS-treated tumors, offering visual proof that DBS effectively initiates adaptive anti-tumor immunity. This immune activation was manifested by heightened infiltration of CD8^+^ cytotoxic T cells and CD4^+^ helper T cells into the TME. Building upon above results, DBS has been shown to effectively hydrolyze collagen in tumor tissues. This collagenolysis results in a loosened tumor matrix, thereby ameliorating the intrinsic high interstitial pressure common in tumors. Such alteration of the TME is strongly correlated with improved T cell infiltration. Flow cytometry analysis further supported these observations, demonstrating a significant increase in the proportions of both CD4^+^ and CD8^+^ T cells in the DBS-treated tumors compared to other groups (Fig. S28). Additionally, flow cytometric analysis of spleen T-cell populations (Fig. 4k–m) showed increased proportions of both CD4^+^ and CD8^+^ T cells in the DBS-treated group. This suggests that DBS not only enhances local intratumoral immunity but also activates systemic immune priming in secondary lymphoid organs, thereby boosting overall antitumor immunity. In brief, our results reveal that DBS induced a more potent anti-tumor immune response than BS. As previous results demonstrated that DBS induces more pronounced maturation of antigen-presenting cells (APCs), it facilitates more efficient presentation of tumor antigens to T cells, ultimately leading to enhanced T-cell infiltration within the tumor. Collectively, these findings demonstrate that DBS significantly potentiated T cell-mediated anti-tumor immunity, offering a promising therapeutic strategy for cancer treatment by transforming the tumor landscape into a more immunologically active and anti-tumor receptive environment, ultimately converting ‘cold’ tumors into ‘hot’ tumors [40].

In conclusion, DBS elicited a coordinated antitumor immune response by promoting DCs maturation, driving macrophage polarization towards the M1 phenotype, and increasing the recruitment and intratumoral infiltration of both CD4^+^ and CD8^+^ T cells. DBS treatment also led to a notable expansion of splenic T cells, indicating effective systemic immune priming. Together, these findings demonstrate that DBS not only reprograms the immunosuppressive tumor microenvironment but also activates systemic immunity, supporting its potential as a robust and multifaceted microbial immunotherapy.

### 2.6 Antitumor effects mediated by intravenous administration of DBS

Given that intratumoral injection of DBS has achieved significant antitumor effects, favorable safety profile, and remarkable immune activation, we further studied the antitumor effects of DBS through intravenous administration. The primary advantage of intravenous delivery for cancer therapeutics lies in its systemic coverage capability, particularly for cancer types that are difficult to treat with in situ injection and are prone to metastasis. This delivery route enables therapeutic agents to reach both primary tumors and distant metastatic sites, in contrast to the localized action of intratumoral injections. Originated from a facultative anaerobic bacterium with an inherent tropism for hypoxic environments, DBS has the potential to specifically target tumor.

**Fig. 5:**
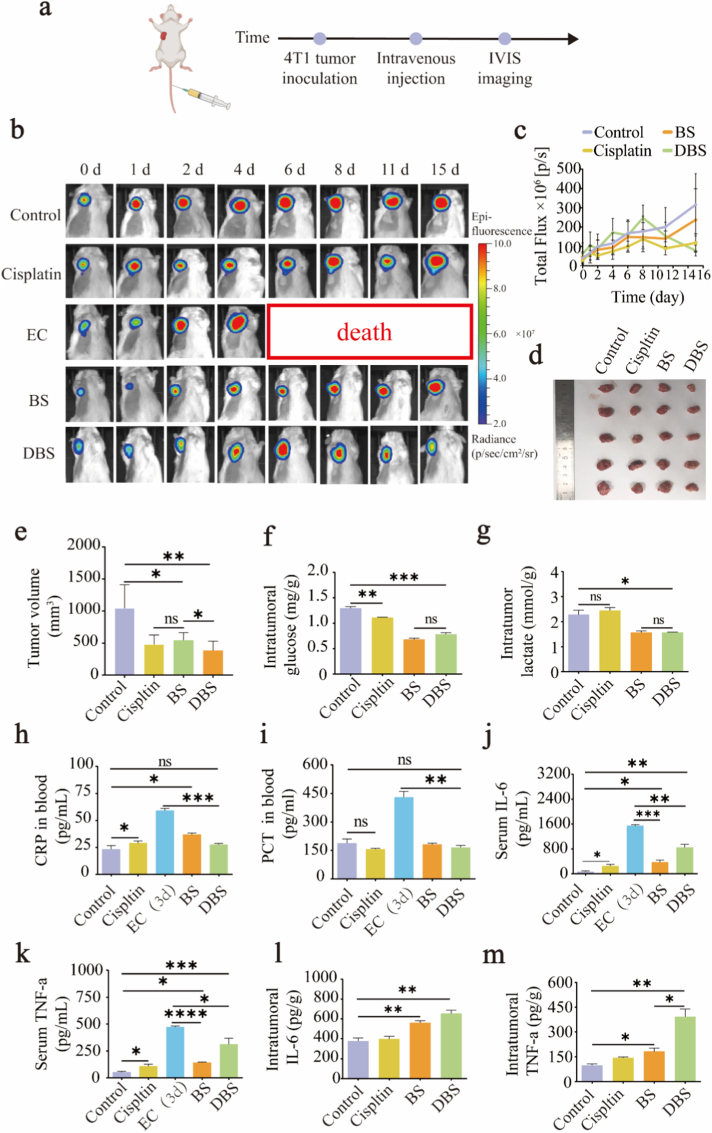
***In vivo* antitumor efficacy, safety evaluation, and TME modulation of BS and DBS following tail vein injection.** a) Experimental design of the tumor treatment via tail vein injection. b) Representative bioluminescence images of tumor-bearing mice after intravenous injection of various therapeutic regimens, with tumors expressing luciferase for visualization. c) Quantification of bioluminescence signals over time. Data are presented as mean ± standard deviation (SD), *n* = 5. d) Image of excised tumors following intravenous therapy. e) Quantification of tumor volume after therapy, presented as mean ± SD (*n* = 5). f) Intratumoral glucose levels after intravenous therapy (mean ± SD, *n* = 3). g) Intratumoral lactate levels after intravenous therapy (mean ± SD, *n* = 3). h) Serum C-reactive protein (CRP) levels after intravenous injection. i) Serum procalcitonin (PCT) levels after intravenous injection (mean ± SD, *n* = 3). j) Serum IL-6 levels after intravenous injection (mean ± SD, *n* = 3). k) Serum TNF-α levels after intravenous injection (mean ± SD, *n* = 3). l) Intratumoral IL-6 levels after intravenous therapy (mean ± SD, *n* = 3). m) Intratumoral TNF-α levels after intravenous therapy (mean ± SD, *n* = 3).

Following intravenous administration of the same therapeutic dose (Fig. 5a), we evaluated the *in vivo* anti-tumor efficacy of DBS. Bioluminescence measurements demonstrated tumor suppression in cisplatin, BS, and DBS groups (Fig. 5b and c), consistent with previous observations after intratumoral injection. Subsequently, we assessed tumor volumes, revealing that treatment with cisplatin, BS, and DBS significantly reduced tumor volumes compared to the control (Fig. 5d and e). Notably, the DBS treatment group exhibited the strongest tumor suppression, showcasing a statistically significant difference compared to the BS group. To verify the metabolic activity of bacteria within tumors and to clarify their tumor-killing mechanisms, we further examined tumor tissues. BS and DBS-treated tumors showed a significant decrease in intratumoral glucose and lactate levels (Fig. 5f and g), indicating that intravenously-administered BS and DBS actively consume key metabolites in the TME, thereby inducing tumor starvation and contributing to direct tumor cell death.

Concurrently, we conducted a thorough evaluation of the safety profile of intravenous DBS administration. Consistent with intratumoral injection results, no significant body weight loss was observed in the BS and DBS groups throughout the treatment period (Fig. S29). All mice in the BS and DBS groups survived the entire treatment without fatalities, while the EC group experienced 100% mortality within four days. This stark contrast highlights that direct intravenous injection of EC, which contains large amounts of immunostimulants like LPS, triggers substantial systemic side effects [41]. Although *E. coli* is commonly used as an engineering strain for tumor therapy, it is not suitable for high-dose intravenous administration. Our findings demonstrate the superior safety of DBS via intravenous injection, underscoring the benefits of leveraging BS as an engineered therapeutic agent.

To further elucidate the safety of DBS, we measured systemic inflammatory biomarkers, including C-reactive protein (CRP) and procalcitonin (PCT), at the endpoint of observation (Fig. 5h and i). Systemic inflammatory responses in the DBS groups showed minimal variation, significantly attenuated relative to the EC group. Crucially, the DBS group demonstrated no significant difference when compared to the control, underscoring their favorable *in vivo* safety profiles even through intravenous administration. To evaluate the balance between systemic and intratumoral immune responses, we quantified the pro-inflammatory cytokines IL-6 and TNF-α in both serum (Fig. 5j and k) and tumor (Fig. 5l and m). Serum levels of both cytokines in DBS groups were significantly lower than those in the EC group, suggesting the improved safety profile of DBS, evidenced by milder and more manageable systemic inflammation compared to EC, as indicated by integrated CRP and PCT analysis. In addition, compared to BS, DBS induced stronger intratumoral immune responses, particularly a significantly higher level of TNF-α, suggesting that its superior tumor-targeting capability enables more effective tumor colonization and sustained local immune activation.

Overall, these findings demonstrate that DBS can be safely and effectively administered intravenously to suppress tumor growth. Its therapeutic efficacy arises from multiple synergistic mechanisms, including targeted tumor colonization, localized drug delivery, induction of tumor starvation, remodeling of the TME, and activation of intratumoral antitumor immune responses. Crucially, for traditional drug delivery systems, achieving such multifaceted therapeutic outcomes often necessitates the design of complex and sophisticated delivery vehicles that encapsulate multiple components. In stark contrast, our engineered bacterial vector, DBS, achieves this therapeutic synergy through a simple, one-step engineering process, without relying on complex carrier designs. The capability of DBS to integrate targeted delivery, localized therapy, and immune modulation into a single therapeutic system provides a distinct advantage over traditional synthetic carriers, offering a more streamlined and potentially more effective strategy for cancer treatment.

### 2.7 Intestinal Homing Effect of DBS and improvement of gut microbiota

Previous studies on microbial tumor therapies have primarily focused on their antitumor efficacy, but the effective clearance of bacteria from the host has not been fully addressed. Interestingly, we observed significantly stronger fluorescent signals in the intestine than in other organs after intratumoral administration of DBS. This observation leads us to hypothesize that DBS, as an active microbial therapeutic agent, may migrate from the tumor to the intestine and subsequently be excreted in feces.

**Fig. 6:**
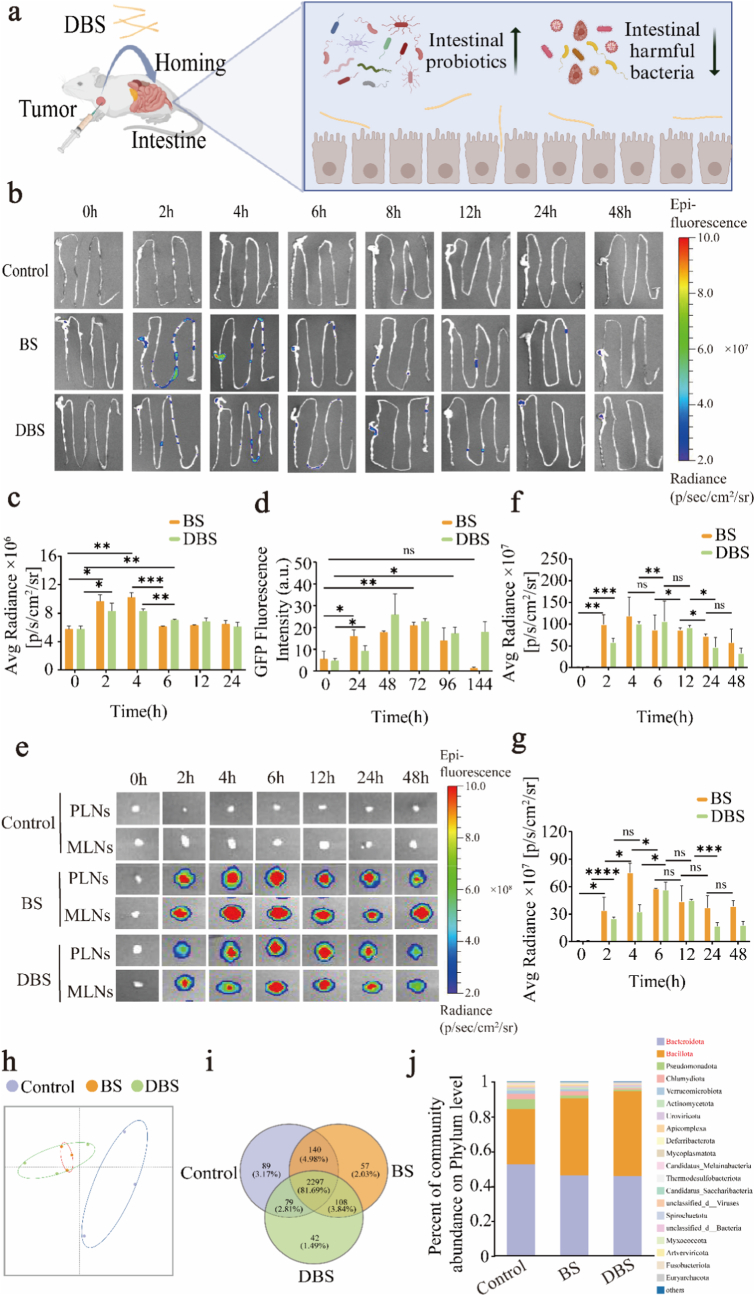
**Intestinal Homing Effect of DBS and Improvement of Gut Microbiota**. a) Schematic illustration of DBS Homing to the gut and improving gut microbiota. b) Representative fluorescence images reflecting changes in gut signals following intratumoral delivery. c) Quantification of gut fluorescence signals in BS-treated and DBS-treated mice d) Fluorescence intensity measurements of intestinal fecal samples taken at different time points post injection and cultured in LB medium, with quantification of BS abundance in fecal samples from BS-treated and DBS-treated mice. e) Representative fluorescence images reflecting changes in nearby tumor-side lymph nodes and mesenteric lymph nodes signals following intratumoral delivery. f) Quantification of PLN fluorescence signals in BS-treated and DBS-treated mice. g) Quantification of MLN fluorescence signals in BS-treated and DBS-treated mice h) PCA analysis indicating community differences across BS, DBS, and control groups. i) Venn diagram illustrating microbial changes post-DBS treatment. j) Community barplot analysis presenting relative abundance changes. All quantified results are expressed as mean ± SD (*n* = 3, biological triplicate).

To further investigate the potential migration of bacteria to the intestine, we examined bacterial fluorescence in isolated intestinal tissues at various time points following intratumoral injection of BS-GFP and DBS-GFP (Fig. 6b). Our quantitative results revealed a significant increase in intestinal fluorescence at both 4 and 6 h post-injection, indicating bacterial translocation into the gut lumen (Fig. 6c). Both the BS and DBS groups exhibited a comparable trend of migration to the intestine. However, the BS group displayed slightly higher peak fluorescence intensity compared to the DBS group. This suggests that the elongated DBS morphology enhances retention within tumors, thereby delaying the translocation of bacteria to the intestine. This finding correlates with the previously observed superior tumor-site retention of DBS.

As the bacteria translocated to the intestine, we next investigated the ultimate fate of the bacteria—specifically, whether the BS or DBS were subsequently cleared from the body through feces. Therefore, fecal samples were collected from each group at predefined time points post-treatment. These samples were cultured and analyzed using flow cytometry to quantify the BS or DBS fluorescence signal (Fig. S33). Analysis of fecal samples showed that fluorescence intensity associated with both BS and DBS first rose within the initial 0-48 h and subsequently declined over time, indicating a period of significant presence and eventual clearance from the gastrointestinal tract (Fig. 6d). This observation confirms that following intratumoral injection, BS and DBS successfully translocated to the intestine and were subsequently eliminated via the fecal route. Considering the strong tumor retention of DBS, it is unlikely that they traveled through the bloodstream. We therefore speculate that translocation to the intestine may have occurred through the lymphatic route. Therefore, we measured the fluorescence intensity in isolated peri-tumoral lymph nodes (PLNs) and mesenteric lymph nodes (MLNs) at various time points following intratumoral injection of Cy5-labeled BS and DBS (Fig. 6e). Our results demonstrated a progressive increase in fluorescence within the PLNs post-treatment, which was subsequently followed by a gradual rise in MLN fluorescence. The increase in MLN fluorescence intensity exhibited a significant lag when compared to PLNs, indicating that intratumorally injected bacteria were transported via lymphatic circulation, moving from the peri-tumoral lymph nodes to more distal lymph nodes (Fig. 6f and g). CFU analysis after intratumoral injection (Fig. S34) showed that the tumor retained a high bacterial load initially, while viable bacteria appeared in the popliteal lymph node (PLN) and mesenteric lymph node (MLN) at 3 h, followed by robust colonization of the small intestine, cecum and feces at 6 h. Only minimal CFU were recovered from blood, liver and spleen, indicating limited systemic spread. This sequential appearance of bacteria (tumor-PLN-MLN- intestine) confirms the lymphatic route of DBS migration, and explains the intestinal fluorescence observed *in vivo*. Bacterial translocation, defined as the movement of bacteria from the intestinal lumen across the intestinal barrier into the systemic circulation or other tissues, is a well-established physiological process [42]. However, in the context of microbial cancer therapy, the reverse process—the migration of therapeutically administered bacteria from the tumor back to the intestine—remains poorly elucidated. We hypothesize that this phenomenon may arise from two complementary factors. Firstly, the intrinsic homing behavior of the bacterial strain. BS is a gut-associated commensal, and its natural compatibility with the intestinal environment likely contributes to its ability to ‘home’ back to the gut after completing its therapeutic functions. This inherent homing tendency provides an important safety advantage by directing the bacteria toward a physiologically familiar and immunologically tolerogenic compartment, thereby minimizing the risk of unintended systemic spread, chronic inflammation, or off-target effects. Secondly, the phagocytes-facilitate transportation. This inflammatory milieu likely promotes the recruitment and activation of immune cells, such as macrophages capable of phagocytosing bacteria or their components. It is plausible that these immune cells, during subsequent trafficking, transport internalized bacteria or bacterial fragments toward lymphoid tissues connected to the gut, ultimately facilitating microbial return to the intestinal tract. Alternatively, residual viable bacteria may escape from the remodeled TME and migrate along lymphatic or circulatory routes that naturally interface with the gut.

As the origin from of DBS, the probiotic BS can coexist with the microbes within the gut, where it promotes the growth of beneficial microbiota, inhibits pathogenic organisms, and enhances immune function [43]. We hypothesize that gut-residing DBS may further modulate intestinal microecology and contribute to the improvement of gut microbiota (Fig. 6a). To explore whether intestine-translocated DBS improve gut microbiota through metabolic interactions, we conducted metagenomic analyses of the intestinal microbiota in mice post-treatment (Fig. 6h-j). Results demonstrated that DBS administration led to marked shifts in microbial composition, showcasing notable increases in the beneficial microbiota phyla such as *Bacillota* and *Bacteroidota* compared to controls. These phyla are recognized for their roles in complex carbohydrate metabolism and regulating short-chain fatty acid production, processes that support gut homeostasis and ultimately contribute to systemic immunity [44]. Conversely, a decrease was observed in potentially pathogenic phyla, including *Pseudomonadota* and *Chlamydiota*. Moreover, the improvement of the gut microbiota may exert an indirect modulatory effect on host immune function [45].

In summary, our findings establish DBS as a highly promising, gut-tropic microbial therapeutic agent with superior safety profile. After fulfilling its anti-tumor function, DBS exhibits gut-homing capability, ensuring efficient clearance via the gastrointestinal tract without causing detectable toxicity. Furthermore, beyond its direct oncolytic activity, DBS presents a multifunctional therapeutic profile, showing potential to actively modulate gut microbiota composition by promoting the proliferation of beneficial commensals. This microbiota normalization is hypothesized to further synergize with its therapeutic effects by enhancing systemic host immune function.

## 3 Conclusion

In conclusion, this work demonstrates that DBS functions as a bacterial therapeutic agent, exerting significant anti-cancer effects through multiple complementary mechanisms, including targeted cisplatin delivery, extended retention, induction of tumor starvation, robust immune activation, and extensive remodeling of the tumor microenvironment. Specifically, DBS serves as a living drug delivery system by exploiting its deformability to efficiently carry cisplatin. Through the targeting of hypoxic tumors, DBS locally release cisplatin within the TME, thereby maximizing drug accumulation at the tumor site while minimizing systemic exposure. This targeted drug delivery is further synergized by DBS-mediated glucose depletion, effectively depriving cancer cells of their primary energy source and rendering them more susceptible to chemotherapy. Furthermore, DBS powerfully reshapes the immune landscape, transforming an immunosuppressive TME into an immune-permissive niche. This deformability-enhanced immune activation is characterized by enhanced DCs maturation, polarization of macrophages towards the pro-inflammatory M1 phenotype, and robust infiltration of CD8^+^ cytotoxic T cells and CD4^+^ helper T cells. DBS actively remodels the physical and metabolic properties of the TME achieved by protease secretion to degrade extracellular matrix components that is the significant barrier to drug delivery and immune cell infiltration, as well as lactate metabolism to mitigate immune evasion and generate a more conducive environment for effector cells. Importantly, after fulfilling its therapeutic functions, DBS demonstrates a superior safety profile by homing to the intestine, facilitating clearance through the gastrointestinal tract, and concurrently reshaping the gut microbiota to favor beneficial phyla while reducing pathogenic ones.

Collectively, our work highlights the translational potential of engineering bacterial morphology for oncolytic microbial therapies. By integrating targeted drug delivery, metabolic interference, immune activation, and TME remodeling into a single platform, DBS exemplifies a powerful and clinically promising approach for next-generation cancer treatment.

## 4 Materials and methods

### 4.1 Bacteria and cell culture

*Bacillus subtilis* strain 168 (wild type), *Bacillus licheniformis* ATCC 14580, *Enterococcus faecalis* OG1RF, and *Escherichia coli* BL21 (DE3) were cultured in lysogeny broth (LB) medium at 37 °C with shaking at 200 rpm. *Pediococcus acidilactici* DSM 20284 and *Lactobacillus bulgaricus* ATCC 11842 were cultured in De Man, Rogosa, and Sharpe (MRS) medium at 37 °C with shaking at 200 rpm.

4T1 mouse breast cancer cells, obtained from our laboratory stock, were cultured in Roswell Park Memorial Institute (RPMI) 1640 medium supplemented with 10% fetal bovine serum (FBS) and 1% penicillin-streptomycin solution (100 U/mL penicillin and 100 μg/mL streptomycin). The murine macrophage cell line RAW 264.7 was obtained from our laboratory stock. Cells were maintained in Dulbecco's Modified Eagle's Medium (DMEM) supplemented with 10% fetal bovine serum (FBS). These cells were incubated at 37 °C in a humidified atmosphere with 5% CO_2_. All cell lines were passaged regularly, ensuring consistent cell health and phenotype.

### 4.2 Preparation of DBS

BS was inoculated in 5 mL of LB medium and cultured for 24 h. The culture was adjusted to an optical density of OD_600_ = 1, followed by centrifugation at 10,000 rpm for 2 min. The resulting pellet was washed three times with sterile water and resuspended in 1 mL of LB medium supplemented with 120 μg/mL Cisplatin. After incubation for 18 h at 30 °C with shaking at 100 rpm, the bacterial cells were pelleted, washed five times with sterile water, and resuspended in 1 mL of PBS to obtain DBS.

To assess the reversibility of cisplatin-induced filamentation, filamentous DBS (generated by an 18 h exposure to 120 μg/mL cisplatin) was harvested, washed, and resuspended in 5 mL fresh LB broth (without cisplatin). The suspension was incubated at 37 °C with 100 rpm shaking, and aliquots were taken at 0, 6, 12, 24 and 48 h for microscopic observation of the cells.

An overnight culture of wild-type *Bacillus subtilis* (BS) was inoculated into 5 mL LB broth and incubated for 24 h. The culture was adjusted to OD_600_ = 1, then harvested by centrifugation at 10,000 rpm for 2 min. The pellet was washed three times with sterile water and resuspended in 1 mL LB containing either 120 μg/mL 5-fluorouracil (5-FU), 120 μg/mL doxorubicin (DOX), or 120 μg/mL paclitaxel (PTX). The suspensions were incubated at 30 °C with 100 rpm shaking for 18 h. After incubation, the bacteria were collected, fixed, and examined by microscopy to assess cell morphology.

### 4.3 Glucose and lactate metabolism by DBS *in vitro*

BS and DBS were cultured in their respective media until reaching the late-exponential growth phase. Cultures were then harvested and adjusted to an optical density at OD_600_ = 5.0. Cells were pelleted by centrifugation at 10,000 rpm for 2 min. The cell pellets were washed three times with sterile, deionized water, and then resuspended in 100 mL of fresh LB medium, separately. Glucose and lactate were filter-sterilized and added separately to the two flasks to achieve final concentrations of 20 g/L and 5 g/L, respectively. Samples were collected after 0, 3, 6, 9, 12, 24, 48 h and quantitative analysis of glucose and lactic acid was performed using a Shimadzu Prominence LC-20 A high-performance liquid chromatography (HPLC) system (Shimadzu Corporation, Kyoto, Japan). The system consisted of a LC-20AT solvent delivery unit, a SIL-20 A auto-sampler, a CTO-20 A column oven, and a RID-20 A refractive index detector. Analytes were separated on an Aminex HPX-87H, 300 mm × 7.8 mm, 9 μm] column (Bio-Rad Laboratories, Hercules, CA, USA). The column oven temperature was maintained at 55 °C. The mobile phase consisted of 5 mM sulfuric acid (H_2_SO_4_), delivered at a flow rate of 0.6 mL/min. The injection volume was 20 μL. Data acquisition and processing were performed using Shimadzu LabSolutions software.

### 4.4 Assessment of protease secretion and cisplatin loading capacity of DBS

DBS was inoculated into 100 mL of LB medium and incubated at 37 °C with shaking at 200 rpm. , and samples were collected at specified time points (0, 12, 24, 36, 48 h) to measure protease activity using a commercial protease assay kit (BOXBIO, China). The cisplatin loading capacity was assessed in parallel across various strains (ICP), including the baseline *B. subtilis* (BS), *B. licheniformis*, *E. faecalis*, *E. coli*, *P. acidilactici* and *L. bulgaricus.*

### 4.5 In vitro immune activation

RAW 264.7 macrophages were harvested and then seeded into 12-well plates at a density of 1 × 10^5^ cells per well, with 1 mL of culture medium per well. The cells were incubated with bacterial suspensions of either BS or DBS at a cell number ratio of 1:100 (bacteria: macrophages). Lipopolysaccharide (LPS) was used as a positive control at a final concentration of 1 mg/mL. Co-culture was maintained for 12 h. Following incubation, the supernatant was removed, and the cells were washed three times and resuspended with PBS.

For surface marker analysis, cells were stained with the following fluorochrome-conjugated antibodies: *Anti*-F4/80-FITC (Cat# 123,107, 0.25 μg per 10^7^ cells, 100 μL volume), Anti-CD206-APC (Cat# 141,707, 0.25 μg per 10^7^ cells, 100 μL volume), and Anti-CD86-PE (Cat# 105,007, 0.25 μg per 10^7^ cells, 100 μL volume). The antibodies were obtained from BioLegend (USA). Staining was performed according to manufacturer's instructions. Flow cytometry analysis was conducted using a BD Accuri C6 flow cytometer. Data was acquired and analyzed with FlowJo software to determine the phenotypic profile of the macrophages.

### 4.6 Cell phagocytosis and cisplatin release by DBS

Freshly cultured or prepared BS and DBS, were incubated with FITC at a concentration equivalent to 0.1 μg FITC per 10^6^ bacteria. Following incubation at 37 °C for 1 h, the bacteria were washed three times and resuspended in PBS to remove unbound FITC. The labeled bacteria were added to RAW264.7 macrophagesat a ratio of 1:10 (bacteria to macrophages) and incubated for predetermined time (0, 3, and 24 h). At each time point, the culture supernatant was carefully aspirated. Macrophages were gently washed three times with PBS to remove free bacteria and FITC. The cells were then harvested and resuspended in PBS for flow cytometry analysis. FITC fluorescence of the macrophages was measured to assess the extent of bacterial phagocytosis.

### 4.7 Tumor localization *in vivo*

Through genetic modification (Fig. S14 and S15), the PNW33N plasmid encoding constitutive expression of cytoplasmic GFP was introduced into BS, generating GFP-expressing strains (BS-GFP). A total of 10^9^ 4T1-Luc cells were injected into the left axillary region of 8-week-old female BALB/c mice and cultured for 7 days to establish the 4T1-Luc tumor-bearing mouse model. Subsequently, 10^6^ GFP-labeled bacteria (BS-GFP and DBS-GFP) were administered via two different routes: intravenous injection through the tail vein and intratumoral injection. *In vivo* imaging was performed using an *In Vivo* Imaging System (IVIS, PerkinElmer) to assess bacterial localization and accumulation.

A 4T1 tumor-bearing mouse model was established, and the mice were subjected to the following treatments: (i) intratumoral injection of free cisplatin, (ii) intratumoral injection of DBS, (iii) tail-vein injection of free cisplatin, and (iv) tail-vein injection of DBS.6 h after administration, the mice were euthanized by cervical dislocation. The heart, liver, lungs, spleen, tumor, and kidneys were rapidly excised, rinsed in ice-cold PBS, blotted dry, and weighed. Each tissue sample (100 mg) was placed in a tube, added 2 mL of trace-metal-grade concentrated HNO_3_, and digested at 70 °C for 2 h. After cooling, the digests were diluted to 10 mL with ultrapure water, filtered through a 0.22 μm membrane, and analyzed for platinum content by inductively coupled plasma mass spectrometry (ICP-MS).

### 4.8 The anti-tumor effect mediated by tail vein injection of BS and DBS

4T1-Luc tumor-bearing mice were randomly assigned into five experimental groups. Each group received intravenous (tail vein) injections of 100 μL of the following: (1) PBS; (2) cisplatin at a concentration of 200 μg/mL; (3) PBS containing 1 × 10^6^ EC; (4) PBS containing 1 × 10^6^ BS; and (5) PBS containing 1 × 10^6^ DBS. The treatment regimen consisted of injections on day 0, 2, 4, 6, 8, 11, 15. During the treatment period, the tumor growth of mice in all treatment groups was regularly monitored through *in vivo* imaging. Changes in tumor size were quantified using an *in vivo* imaging system (IVIS, Pekin Elmer) by measuring bioluminescence signals emitted from the 4T1-Luc cells. Prior to imaging, mice received an intraperitoneal injection of D-luciferin (150 mg/kg body weight) and were allowed to anesthetize for 10-15 min to ensure optimal substrate distribution and activity. Bioluminescence images were acquired, and data were analyzed using Living Image software. At the conclusion of the treatment period, blood samples were collected from all mice. Plasma levels of inflammatory markers, including C-reactive protein (CRP) and procalcitonin (PCT) were quantified in each sample. Circulating levels of the cytokines interleukin-6 (IL-6) and tumor necrosis factor-alpha (TNF-α) were also measured. Subsequently, mice were euthanized, and tumors were harvested and used to measure levels of IL-6 and TNF-α to assess intratumoral immune activation. ELISA kits for TNF-α, IL-6, PCT, and CRP were purchased from 4 A Biotech., Co. Ltd. (China) and used directly without any further purification.

### 4.9 The anti-tumor effect mediated by intratumoral injection of BS and DBS

Intratumoral injections in tumor-bearing mice were performed under the same experimental conditions as described in section 4.8, with identical antitumor monitoring and ELISA measurements. The collagen content in the tumor tissues was assessed using the QuickZyme Sensitive Tissue Collagen Assay (QuickZyme Biosciences), following the manufacturer's instructions. Additionally, tumor tissues were fixed, embedded, and subjected to histological staining, including hematoxylin and eosin (H&E), Masson's trichrome, and TUNEL staining, conducted by Wuhan ServiceBio Technology Co. Ltd. to evaluate tissue morphology, collagen deposition, and apoptosis.

### 4.10 Immune response activation by DBS *in vivo*

Following three rounds of Intratumoral injection treatment, as described in Sections 4.9, we assessed immune cell populations in the TME and secondary lymphoid organs. Inguinal lymph nodes (draining the tumor) were harvested. Single-cell suspensions were prepared and stained with the following antibodies: Anti-CD11c-APC (CAT# 117,309, 0.25 μg per 10^7^ cells, 100 μL volume), Anti-CD80-FITC (CAT# 104,705, 0.25 μg per 10^7^ cells, 100 μL volume) and Anti-CD86-PE (CAT# 105,007, 0.25 μg per 10^7^ cells, 100 μL volume), to identify mature dendritic cells (DCs) by flow cytometry. Tumor tissues were harvested and dissociated into single-cell suspensions. These cells were stained with the same macrophage antibodies as described in section 4.5 to analyze changes in macrophage phenotype using flow cytometry. Single-cell suspensions were prepared from both tumor tissues and spleens. These cells were stained with the following antibodies: Anti-CD3-APC (CAT# 100,235, 0.25 μg per 10^7^ cells, 100 μL volume), Anti-CD4-FITC (CAT# 100,405, 0.25 μg per 10^7^ cells, 100 μL volume), and Anti-CD8a-Cy5 (CAT# 100,709, 0.25 μg per 10^7^ cells, 100 μL volume) to analyze the percentages of CD4^+^ and CD8^+^ T cells using flow cytometry. All antibodies were obtained from BioLegend (USA).

### 4.11 DBS homing effect and gut microbiota improvement

Tumor-bearing mice received intratumoral injections of either BS-GFP or DBS-GFP. At the following time points post-injection: 0 h, 2 h, 4 h, 6 h, 8 h, 12 h, 24 h, 48 h, 72 h, 96 h, and 144 h, mice were euthanized, and their intestines were harvested and subjected to fluorescence imaging to assess bacterial homing potential. In addition, fecal samples were collected at the same time points. Fecal pellets were resuspended in 1 mL of sterile PBS, and the resulting suspension was centrifuged at 5000 rpm for 5 min 200 μL of the supernatant was then transferred to 1 mL of LB medium and incubated at 37 °C with shaking at 200 rpm for 6 h. After incubation, microbial cells were pelleted via centrifugation at 1000 rpm for 2 min and washed twice with PBS. The resulting pellet was resuspended in 500 μL of PBS, and flow cytometry was performed to analyze GFP fluorescence signals. Simultaneously, fecal samples were sent to Shanghai Majorbio Bio-Pharm Technology Co., Ltd. for metagenomic sequencing. Analysis of the resulting data was used to assess changes in the gut microbiota composition. Tumor-bearing mice were subjected to intratumoral injection of Cy5-labeled BS or DBS. At specified time points post-injection: 0, 2, 4, 6, 12, 24, and 48 h, mice were humanely euthanized. The PLNs and MLNs were then harvested to assess the lymphatic dissemination of the bacteria. Blood samples were collected at treatment completed to assess liver and kidney function by analyzing serum biochemical parameters Alanine Aminotransferase (ALT), Aspartate Aminotransferase (AST), Creatinine (CREA), and Uric Acid (UA) using Biochemistry Analyzer. Major organs (liver, kidney, spleen, heart, lung) were harvested and fixed in 4% paraformaldehyde for subsequent histological examination. Tissues were embedded in paraffin, sectioned, and stained with hematoxylin and eosin (H&E) to assess histocompatibility and potential treatment-related toxicity. Histopathological analysis was performed. All histological assessment was performed by Wuhan ServiceBio Technology Co., Ltd.

In vivo tracking of DBS-GFP migration after intratumoral injection: 4T1 tumor-bearing mouse model was established, and an intratumoral injection of 1 × 10^8^ DBS-GFP was performed. Mice were euthanized by cervical dislocation at 0, 3, 6, 12, and 24 h post-injection. The tumor, popliteal lymph node (PLN), mesenteric lymph node (MLN), small intestine, cecum, feces, serum, liver, and spleen were harvested, weighed, and homogenized in sterile PBS. Homogenates were serially diluted in PBS, and 100 μL of each dilution was spread onto LB agar plates containing chloramphenicol (25 μg/mL) to select for the GFP-expressing strain. Plates were incubated at 30 °C for 12 h. All procedures were performed in triplicate, and data are presented as mean ± SD.

### 4.12 Statistical analysis

Data were presented as mean ± SD. Comparisons between two groups employed Student's t-test and comparisons among multiple groups used one-way ANOVA. Significance levels were denoted by ∗ p < 0.05, ∗∗p < 0.01, ∗∗∗p < 0.001, ∗∗∗∗p < 0.0001, and “ns” stands for “not significant”. GraphPad Prism 9.5 software was utilized for statistical analyses. All experiments were performed in biological replicates (*n* = 3 or *n* = 5).

## CRediT authorship contribution statement

**Tao Sun:** Conceptualization, Data curation, Formal analysis, Methodology, Software, Validation, Writing – original draft. **Xiang Wang:** Formal analysis, Writing – original draft, Writing – review & editing. **Yawen Jiang:** Conceptualization, Investigation, Software, Supervision. **Mingxiu Liu:** Funding acquisition, Investigation, Project administration, Resources, Supervision, Visualization. **Lianting Huang:** Data curation. **Limei Yang:** Funding acquisition. **Bei Guo:** Validation. **Kewei Wang:** Funding acquisition, Project administration, Resources, Writing – review & editing. **Guodong Sun:** Conceptualization, Data curation, Formal analysis, Funding acquisition, Investigation, Methodology, Project administration. **Yi Zhang:** Data curation, Formal analysis, Funding acquisition, Investigation, Methodology, Project administration, Resources, Software, Supervision, Validation. **Wei Xue:** Conceptualization, Data curation, Formal analysis, Funding acquisition, Investigation, Methodology, Project administration, Resources, Software.

## Declaration of competing interest

The authors declare that they have no known competing financial interests or personal relationships that could have appeared to influence the work reported in this paper.

## Data Availability

No data was used for the research described in the article.
