# Supplementary material for: A deformable Bacillus subtilis with gut homing for tumor treatment via bacterial metabolism-facilitated synergistic approach for starvation, chemotherapy, and immunotherapy
Source: Mater Today Bio. 2026 Jun 1;38:103295. doi: 10.1016/j.mtbio.2026.103295 (PMC13255084; doi:10.1016/j.mtbio.2026.103295)
Supplement: Multimedia component 1 [file mmc1.docx]

**A Deformable *Bacillus Subtilis* with gut homing for Tumor Treatment via bacterial metabolism-facilitated Synergistic Approach for** **Starvation, Chemotherapy, and Immunotherapy**

Tao Sun ^a,#^, Xiang Wang ^a,#^, Yawen Jiang ^b,#^, Mingxiu Liu ^e,#^, Lianting Huang ^a^, Limei Yang ^a^, Bei Guo ^a^, Kewei Wang ^a,^ *，Guodong Sun ^c^ *, Yi Zhang ^a,c,d^ *, Wei Xue ^a,d,^*

a *Guangdong Provincial Engineering and Technological Research Center for Drug Carrier Development, Department of Biomedical Engineering, Jinan University, Guangzhou 510632, China.*

b *College of Traditional Chinese Medicine, Jinan University, Guangzhou 510632, China*

c *Guangdong Provincial Key Laboratory of Spine and Spinal Cord Reconstruction, The Fifth Affiliated Hospital (Heyuan Shenhe People’s Hospital), Jinan University, Heyuan 517000, China.*

d *MOE Key Laboratory of Tumor Molecular Biology, Jinan University, Guangzhou 510632, China*

*e College of Agricultural and Life Science, University of Wisconsin-Madison*

⁎ Corresponding author.

Kewei Wang: wangkw@jnu.edu.cn

Guodong Sun: sgd96@jnu.edu.cn

Yi Zhang: zhangyi_0424hot@163.com

Wei Xue: weixue_jnu@aliyun.com

# These authors contributed equally to this work.


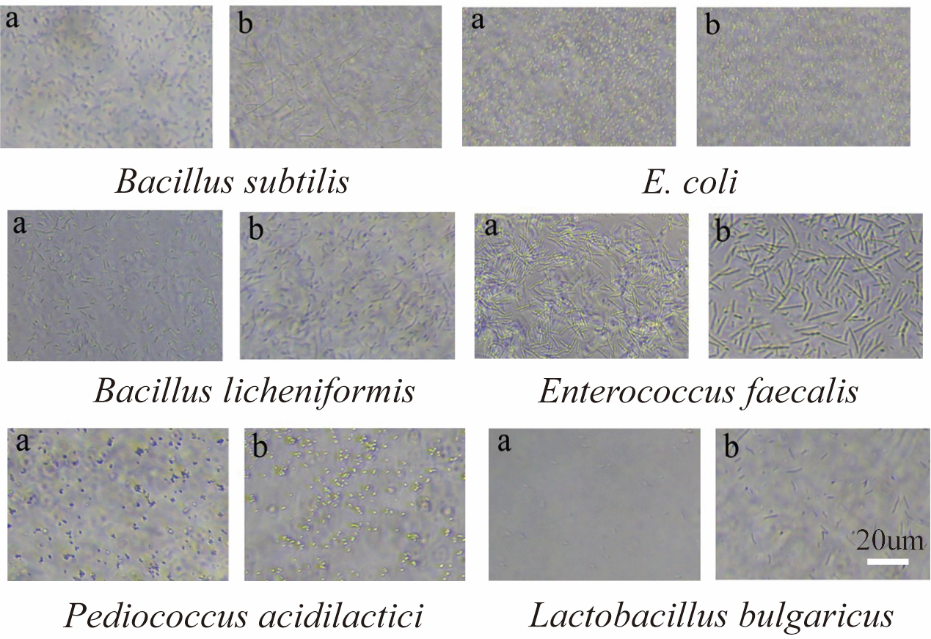


**Figure S1.** Images of the deformation effects of different probiotics under cisplatin induction. a) before induction, b) after induction.


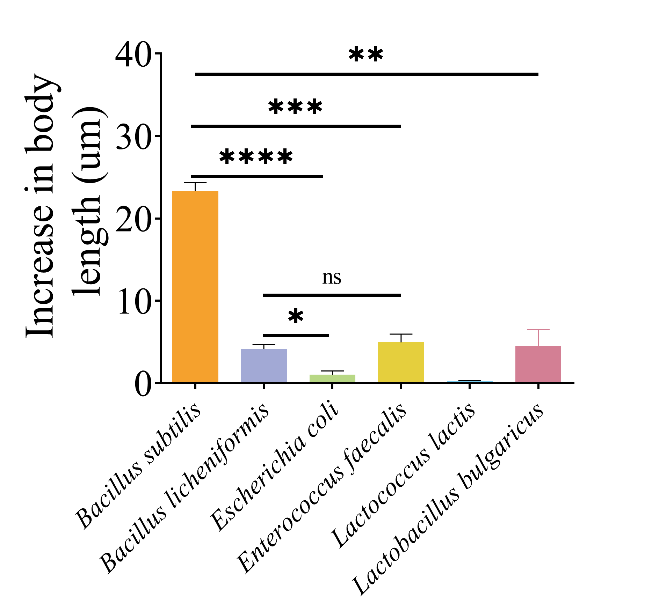


**Figure S2.** Increase in cell lengths of various strains after cisplatin induction.


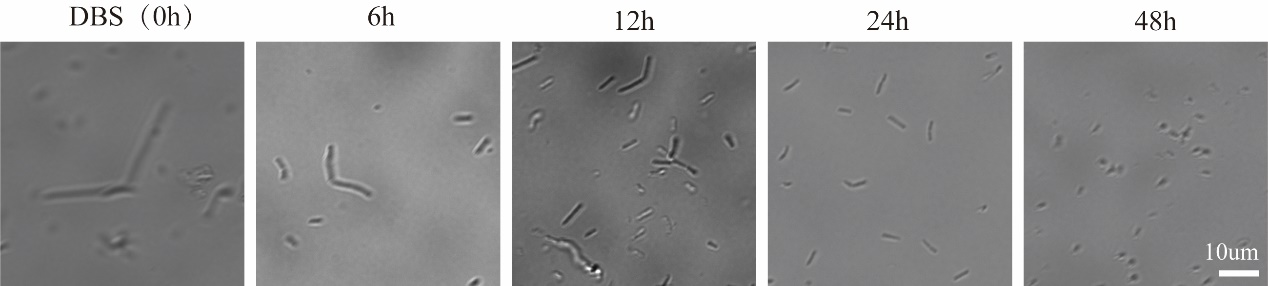


**Figure S3.** Recovery of cisplatin‑induced filamentation in *Bacillus subtilis* after transfer to drug‑free LB medium.

**
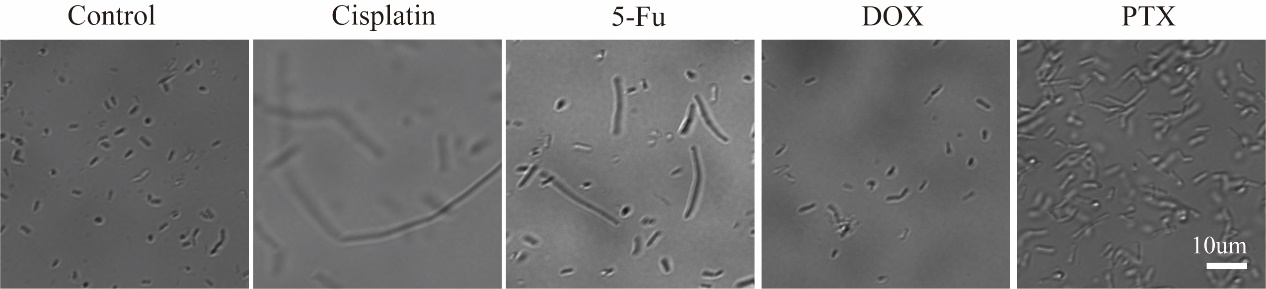
**

**Figure S4.** Morphological response of *Bacillus subtilis* to different anticancer agents.


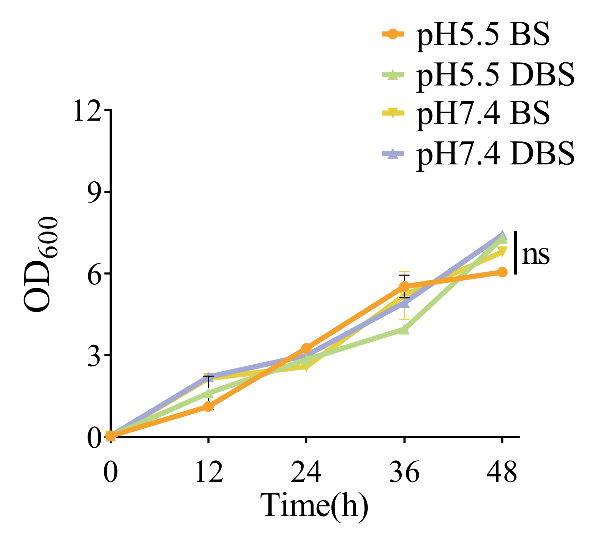


**Figure S5.** Growth Curves of BS and DBS at pH 5.5 and pH 7.4, with quantitative data expressed as mean ± SD (n = 3).


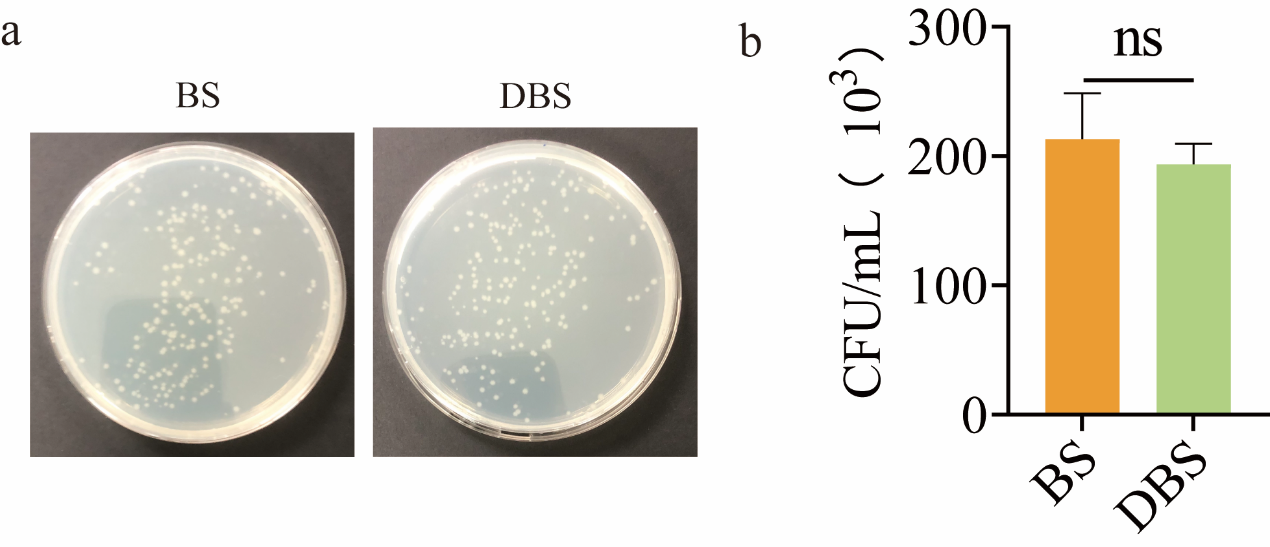


**Figure S6.** Assessment of viability of untreated *B. subtilis* (BS) and cisplatin‑loaded, filamentous *B. subtilis* (DBS). (a) Plate‑growth assay. (b) Quantitative colony‑forming‐unit (CFU) analysis.


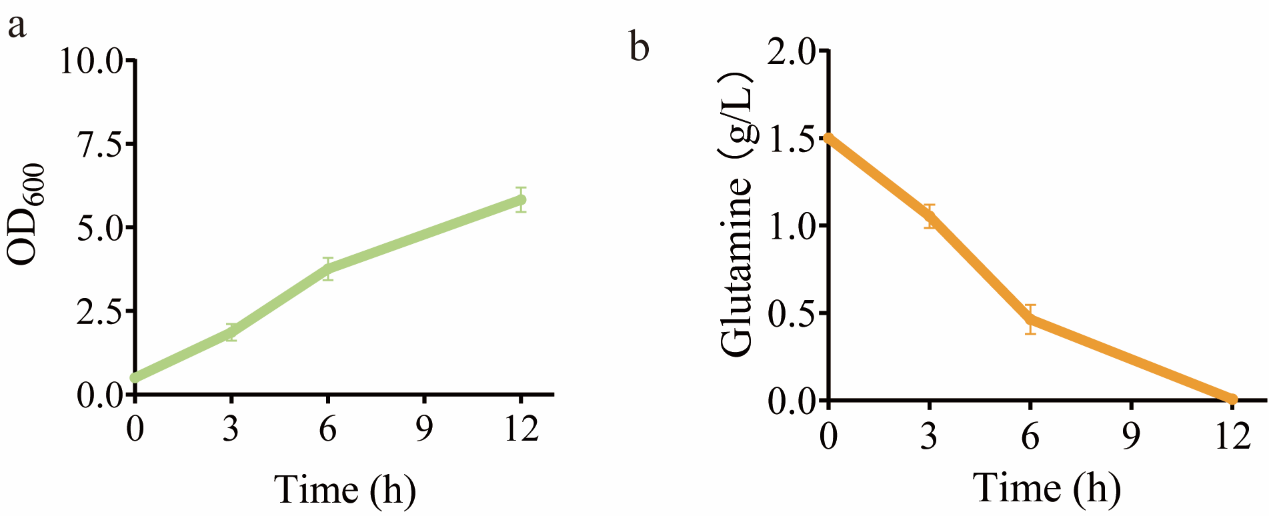


**Figure S7.** Glutamine utilization by *Bacillus subtilis* (BS) in vitro. (a) Growth kinetics of BS cultured in medium with glutamine as the sole nitrogen source. (b) Time‑dependent consumption of glutamine by BS in the same medium.


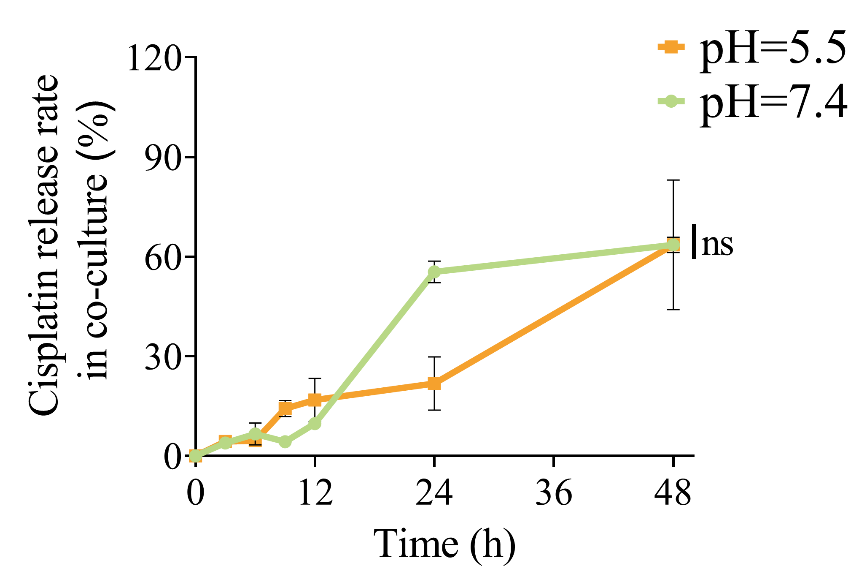


**Figure S8.** Release curves of cisplatin loaded in DBS at different time points under different pH conditions, with quantitative data expressed as mean ± SD (n = 3).


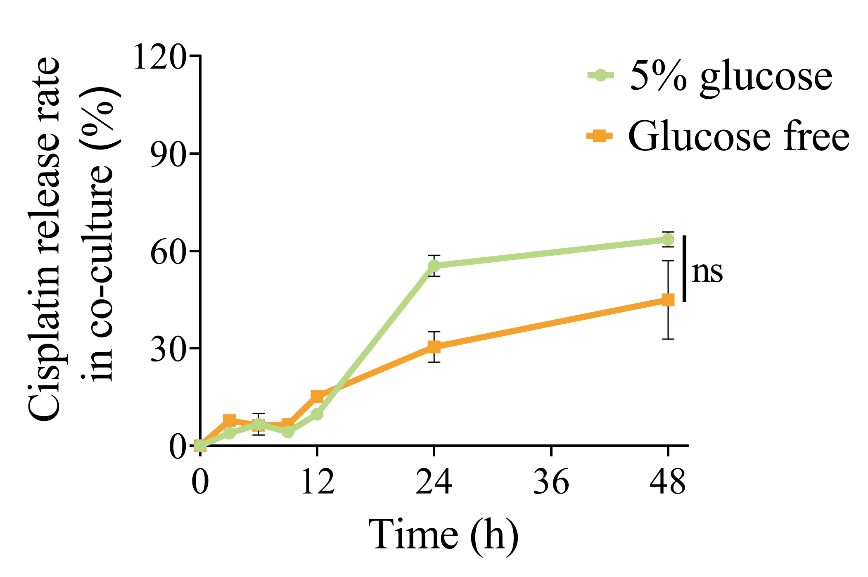


**Figure S9.** Release curves of cisplatin loaded in DBS at different time points under different glucose concentrations, with quantitative data expressed as mean ± SD (n = 3).


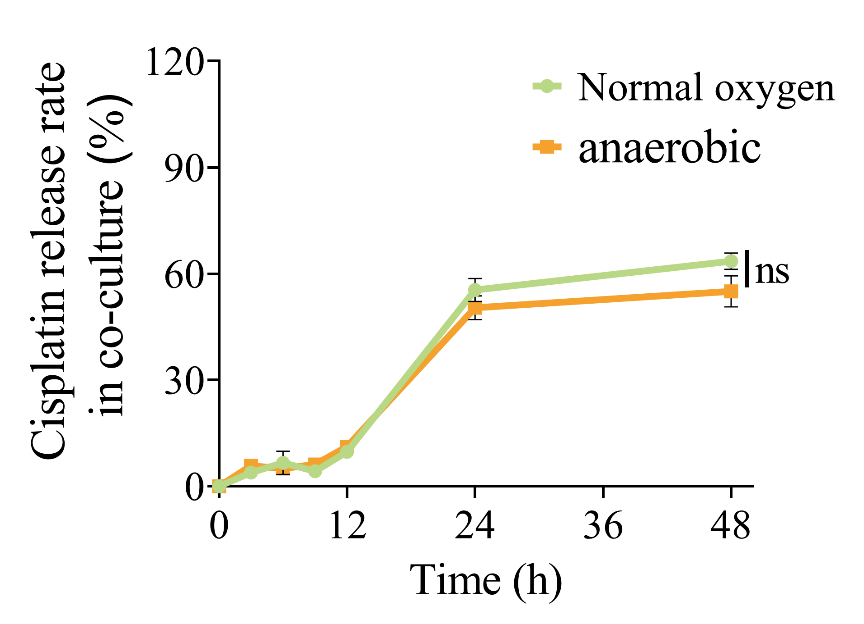


**Figure S10.** Release curves of cisplatin loaded in DBS at different time points under varying oxygen concentrations, with quantitative data expressed as mean ± SD (n = 3).


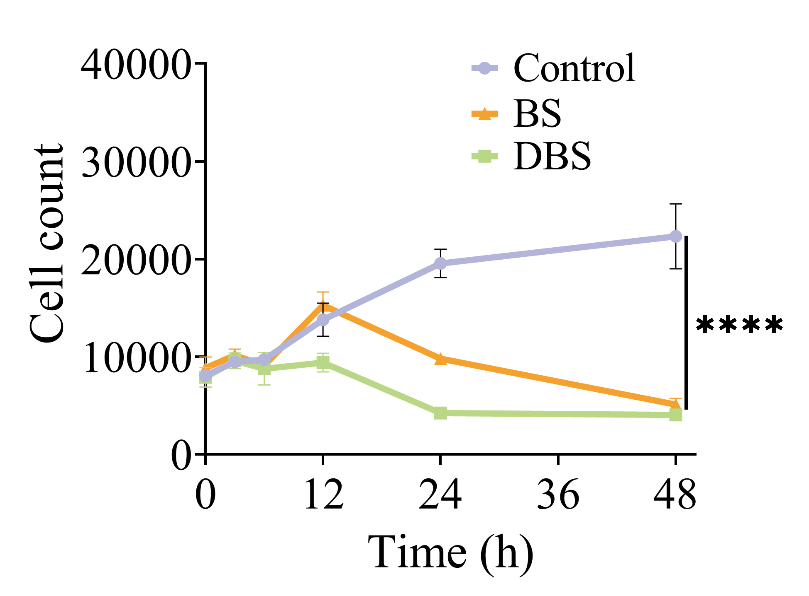


**Figure S11.** Impact of BS and DBS on 4T1 cell proliferation over time, with quantitative data expressed as mean ± SD (n = 3).


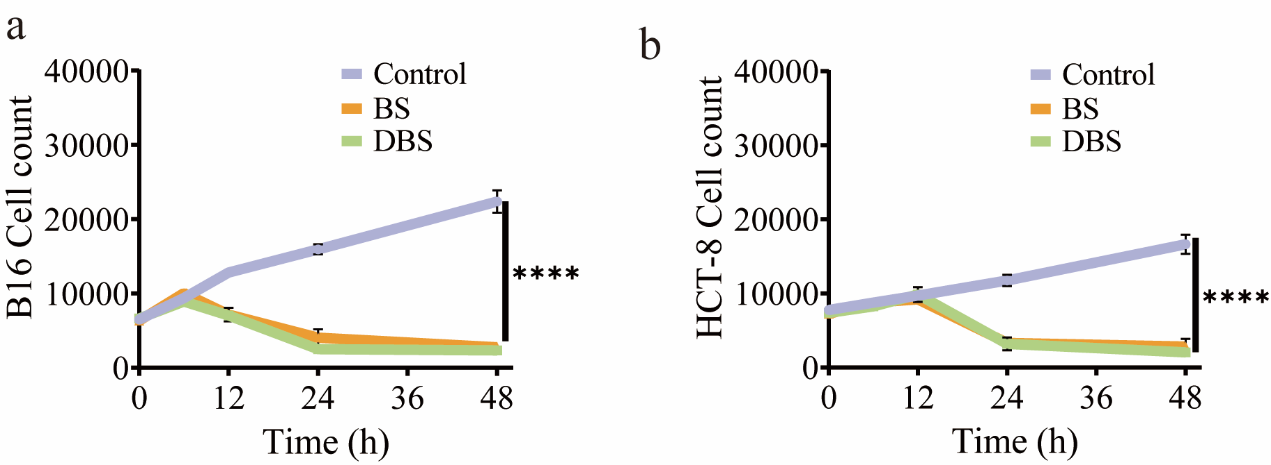


**Figure S12.** Time‑dependent cytotoxicity of DBS against cancer cell lines. (a) B16 melanoma cells，(b) HCT‑8 human colorectal adenocarcinoma cells, with quantitative data expressed as mean ± SD (n = 3).


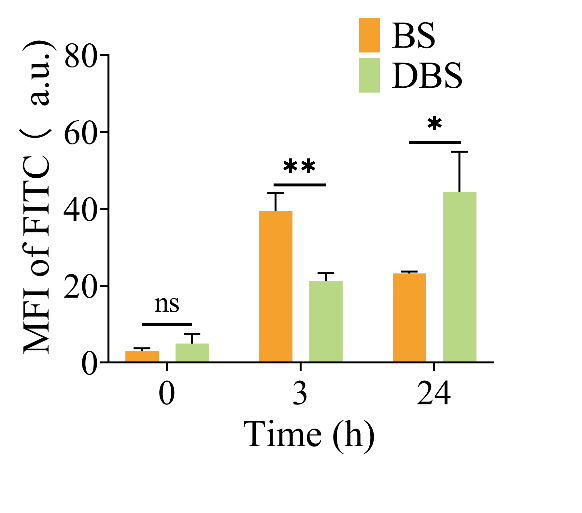


**Figure S13.** m) In vitro assessment of macrophage phagocytosis of BS and DBS. All quantified results are expressed as mean ± SD (*n* = 3, biological triplicate).


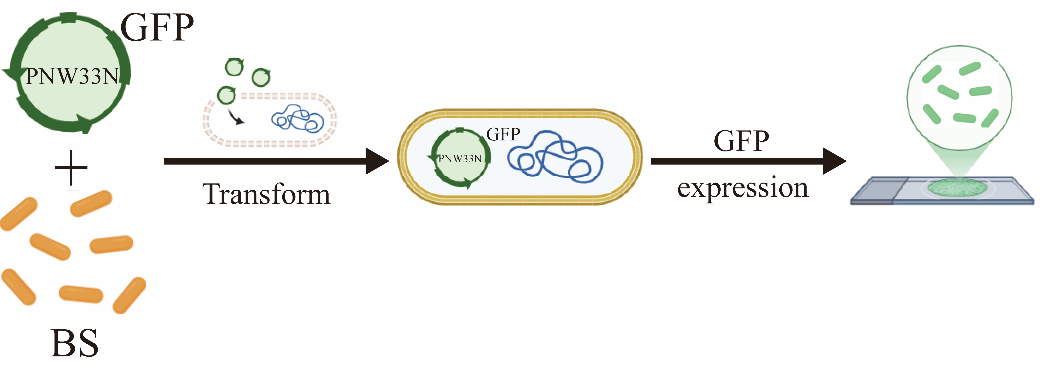


**Figure S14.** Flow chart for the preparation of BS-GFP through genetic engineering.


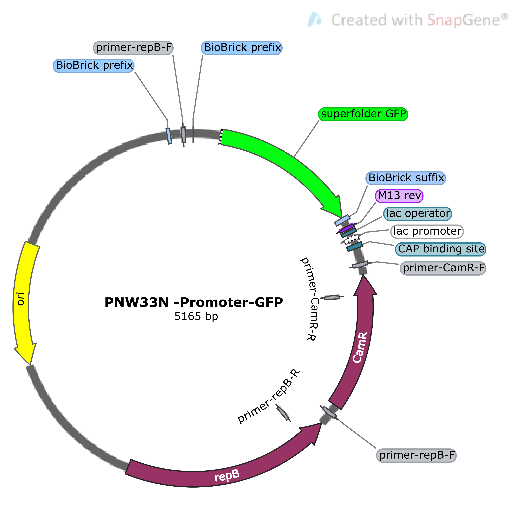


**Figure S15.** Plasmid map of the BS expression vector PNW33N.
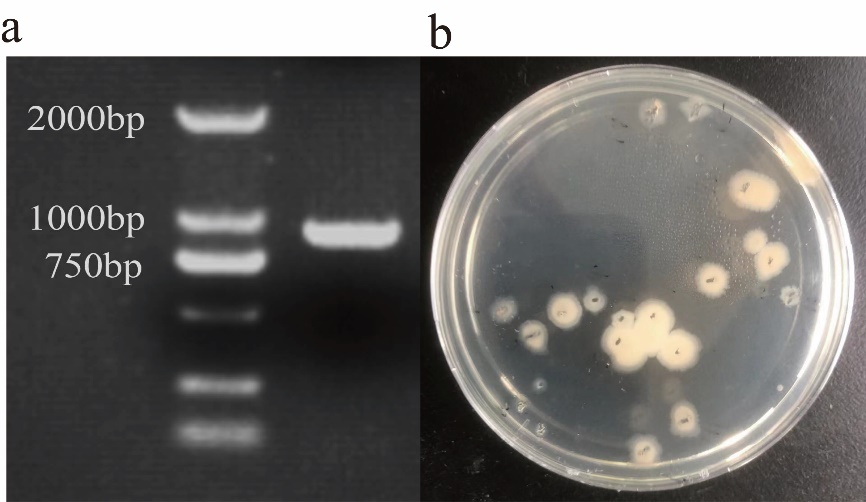


**Figure S16.** a) Agarose gel electrophoresis results for the identification of BS transformed with the PNW33N vector, b) Screening of BS transformed with the PNW33N vector on chloramphenicol selection medium.


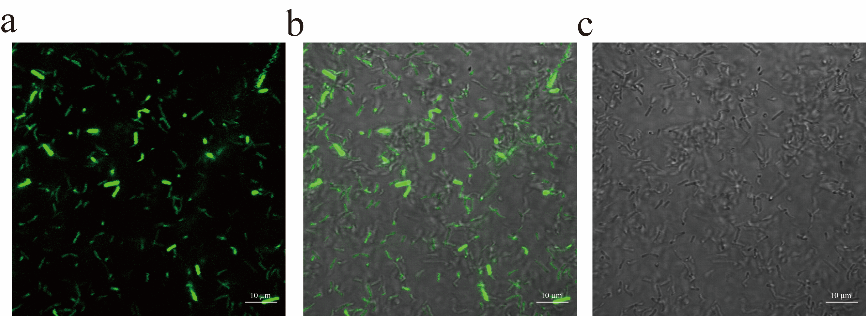


**Figure S17.** Laser confocal images of recombinant bacteria BS-GFP expressing green fluorescent protein: a) Imaging of bacterial luminescence under fluorescence field, b) Imaging of bacterial luminescence under fluorescence and bright field, c) Imaging of bacteria under bright field (Scale bar: 10 μm).

**
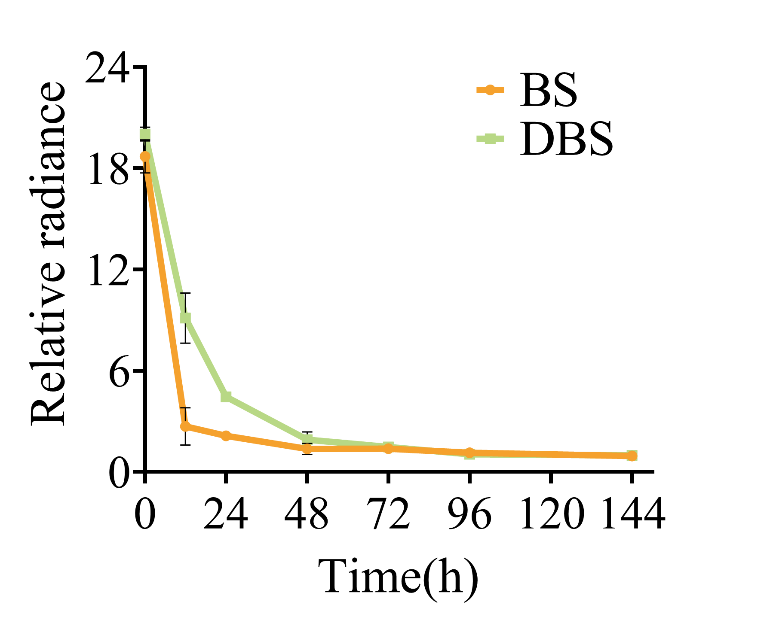
**

**Figure S18.** Relative fluorescence changes in tumors over time post-intratumoral injection. All quantified results are expressed as mean ± SD (*n* = 5, biological replicate).

**
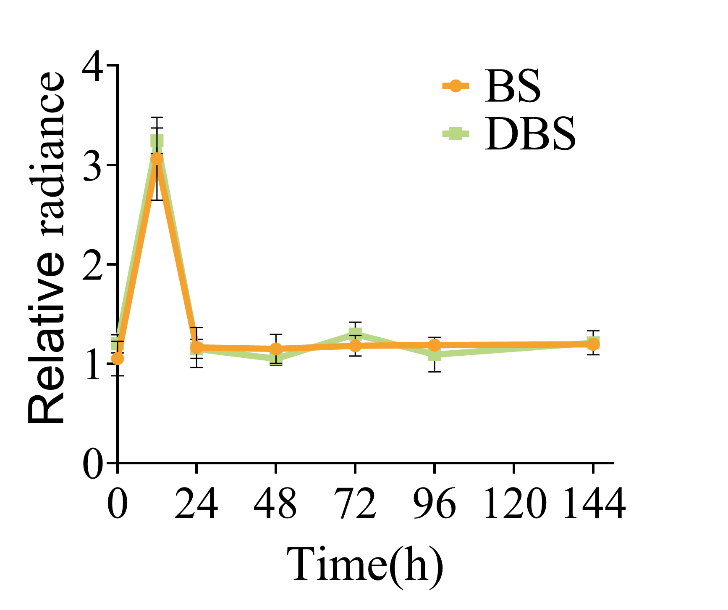
**

**Figure S19.** Relative fluorescence changes in tumors over time post-tail vein injection, all quantified results are expressed as mean ± SD (*n* = 5, biological replicate).


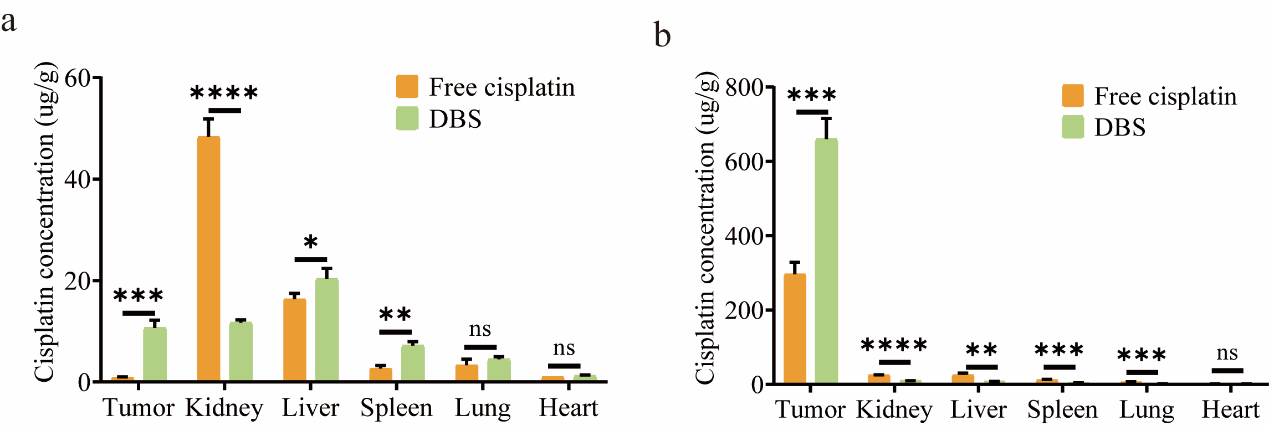


**Figure S20.** Cisplatin content in major organs 6 h after delivery of free cisplatin versus cisplatin‑loaded *Bacillus subtilis* (DBS). (a) Tail‑vein injection. (b) Intratumoral injection.


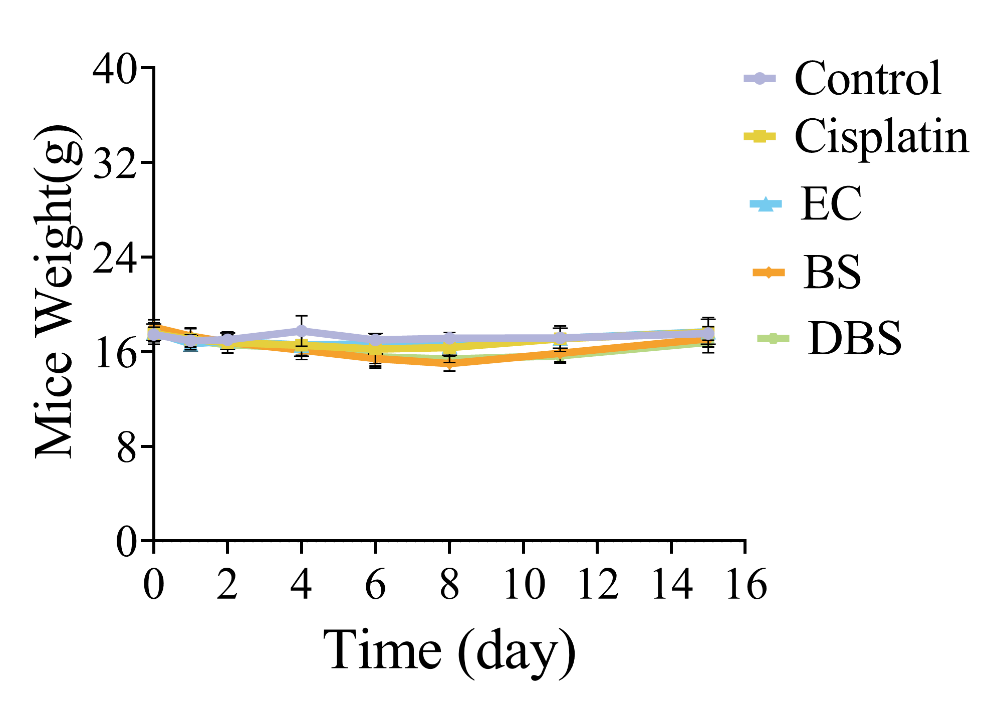


**Figure S21.** Images showing changes in mouse body weight during treatment with different drugs delivered via intratumoral injection, with quantitative data expressed as mean ± SD (n = 5).


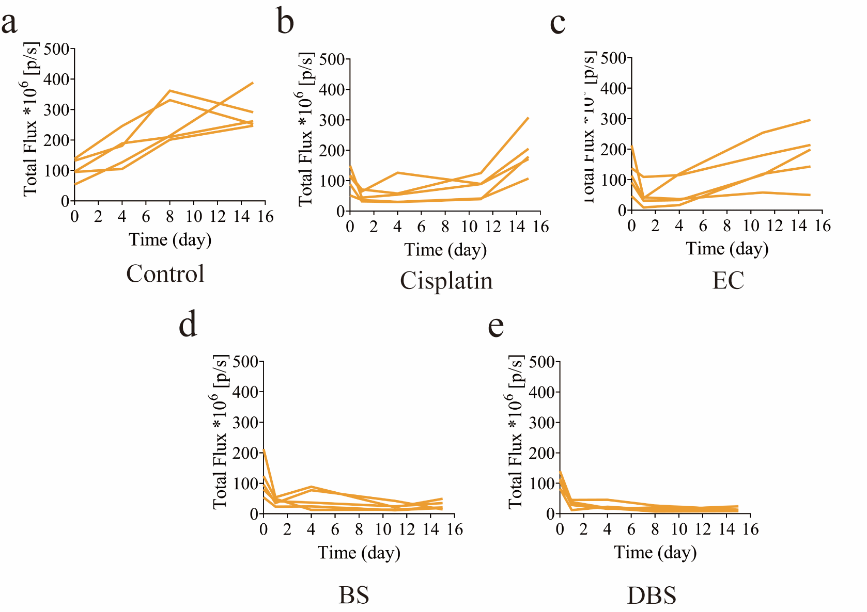


**Figure S22.** Images of individual tumor luminescence at different time points during treatment with different drugs delivered via intratumoral injection: a) Control, b) Cisplatin, c) EC, d) BS, e) DBS, with quantitative data expressed as mean ± SD (n = 5).


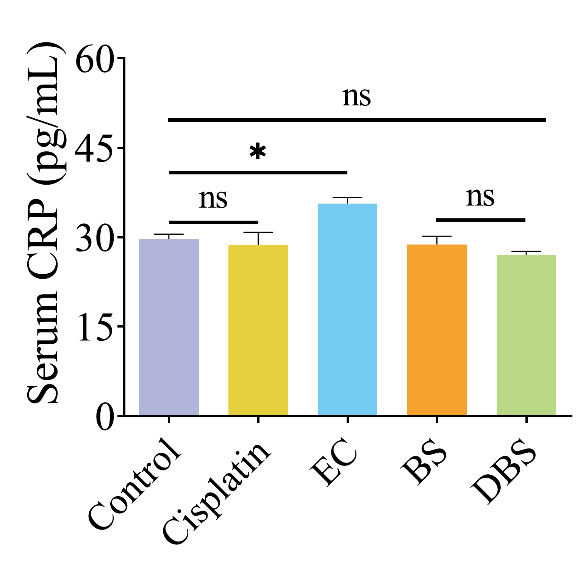


**Figure S23.** Serum C-reactive protein (CRP) levels after intratumoral administration (mean ± SD, *n*=3).

**
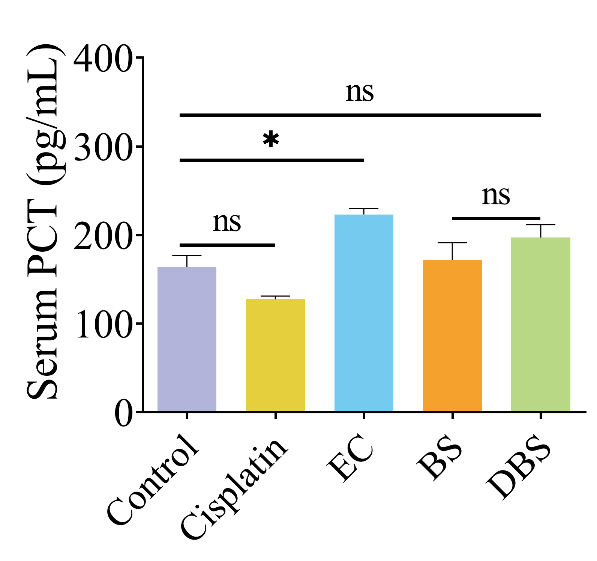
**

**Figure S24.** Serum procalcitonin (PCT) levels after intratumoral administration (mean ± SD, *n*=3).

**
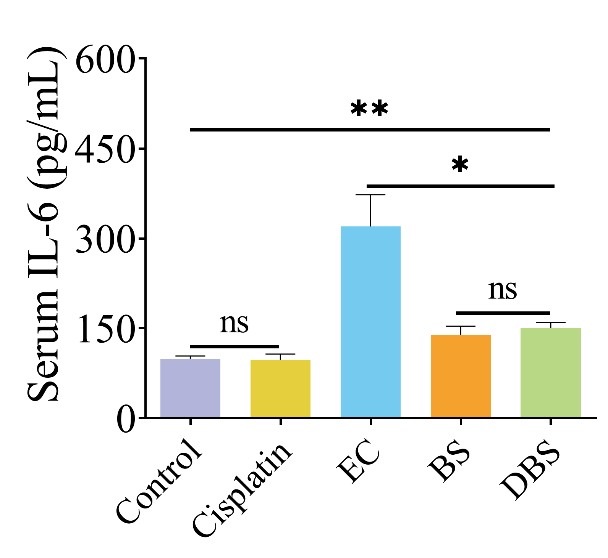
**

**Figure S25.** Serum IL-6 levels after intratumoral administration (mean ± SD, n=3)

**
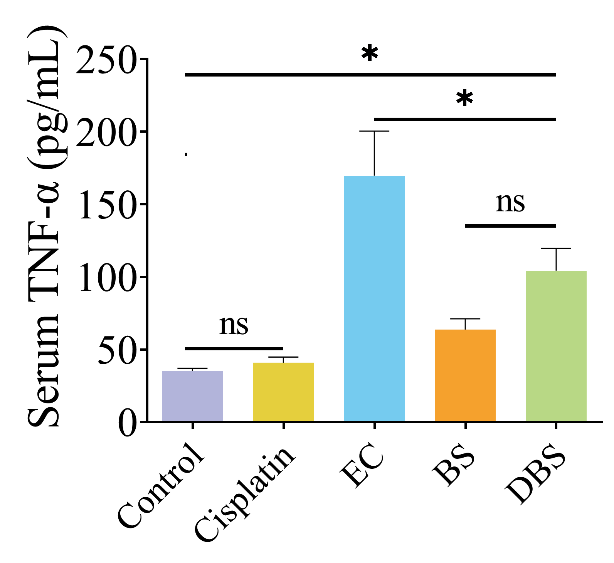
**

**Figure S26.** Serum TNF-α levels after intratumoral administration (mean ± SD, *n*=3).

**
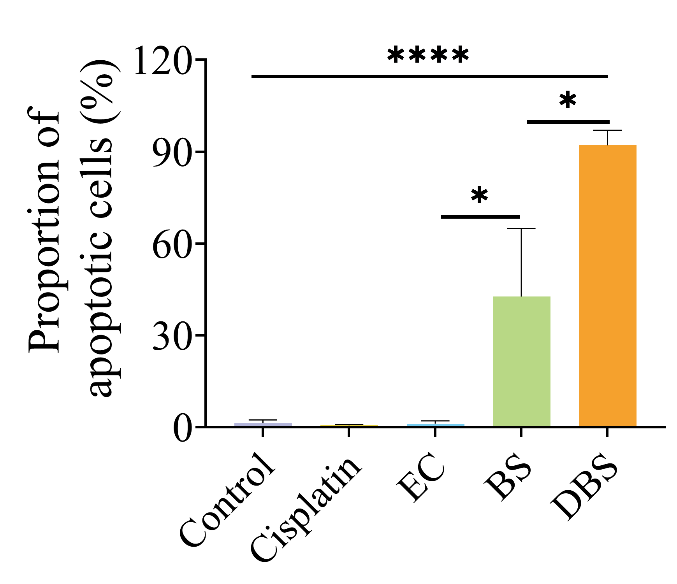
**

**Figure S27.** Quantification of the percentage of TUNEL-positive cells per group by ImageJ analysis. Data are shown as mean ± SEM (n = 3).


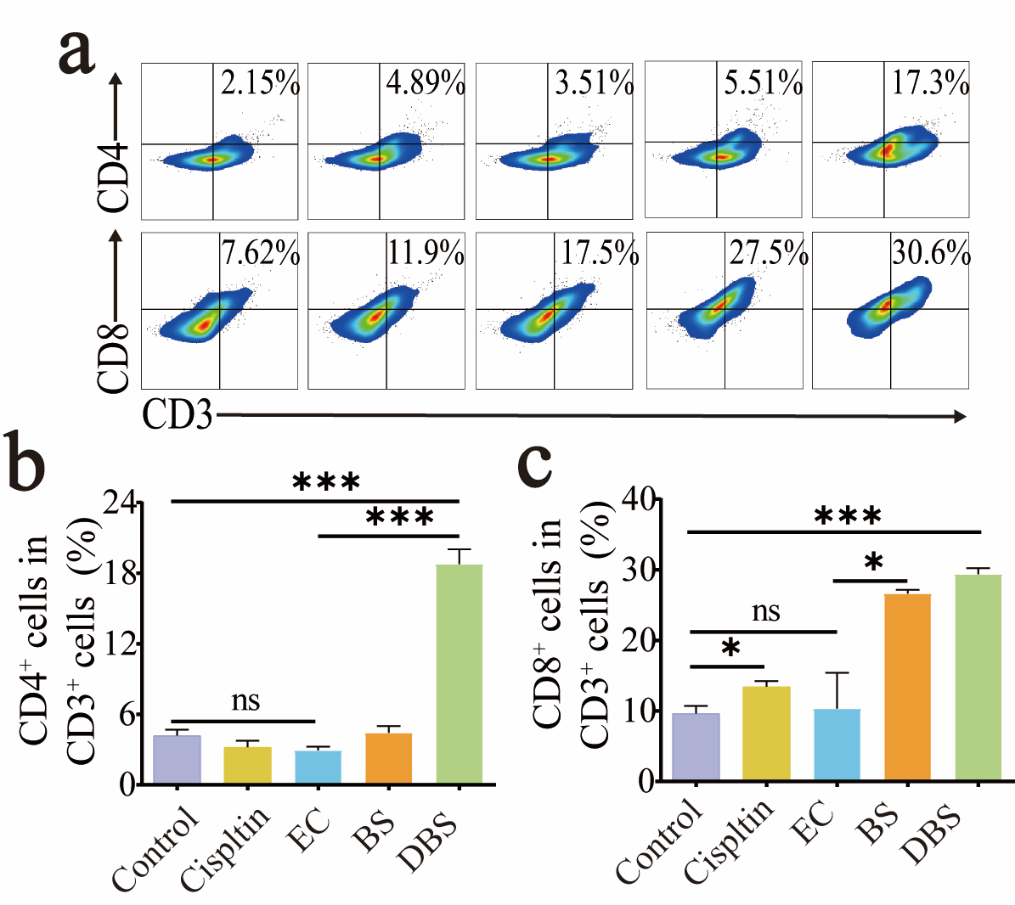


**Figure S28.** a) Representative flow cytometry analysis of CD4^+^ and CD8^+^ phenotypes in T cells (CD3^+^) in tumor tissue after intratumoral therapy and the quantitative percentage of CD4^+^ T cells h) and CD8^+^ T cells b). c)


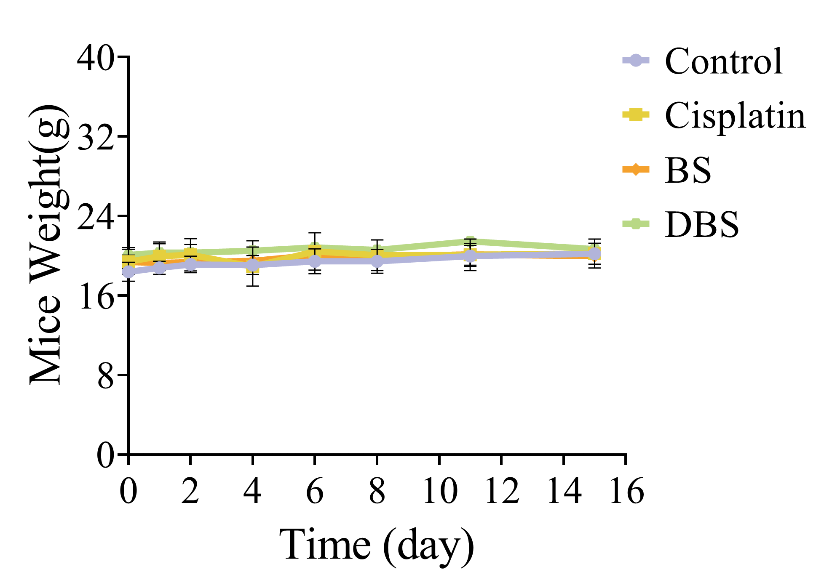


**Figure S29.** Images showing changes in mouse body weight during treatment with different drugs delivered via tail vein injection, with quantitative data expressed as mean ± SD (n = 5).


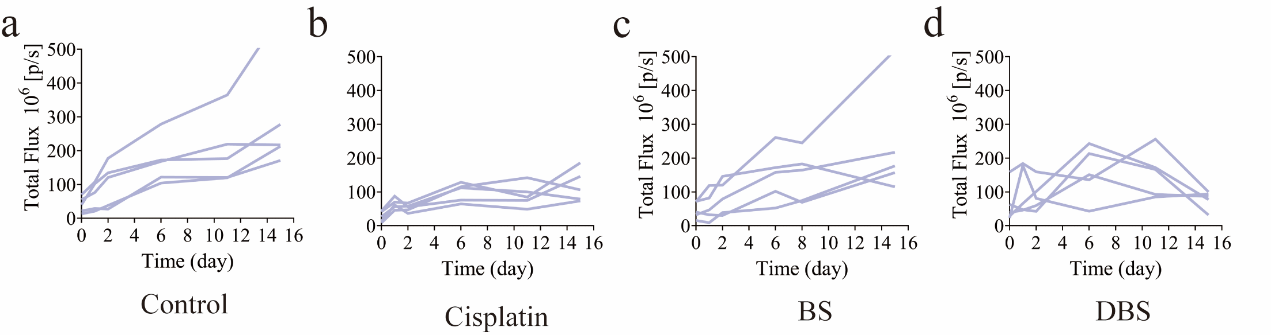


**Figure S30.** Images of individual tumor luminescence at different time points during treatment with different drugs delivered via tail vein injection: a) Control, b) Cisplatin, c) BS, d) DBS, with quantitative data expressed as mean ± SD (n = 5).


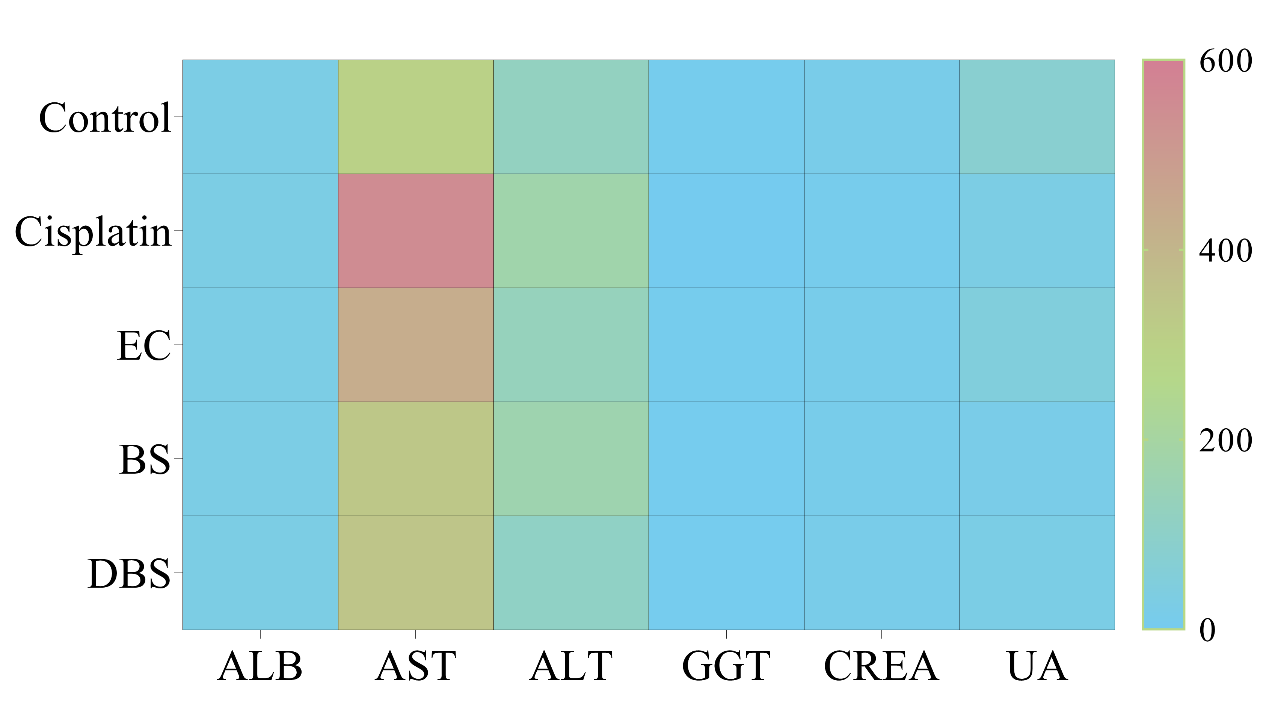


**Figure S31.** Heatmap analysis of liver and kidney function in blood post-treatment with different drugs delivered via intratumoral injection.


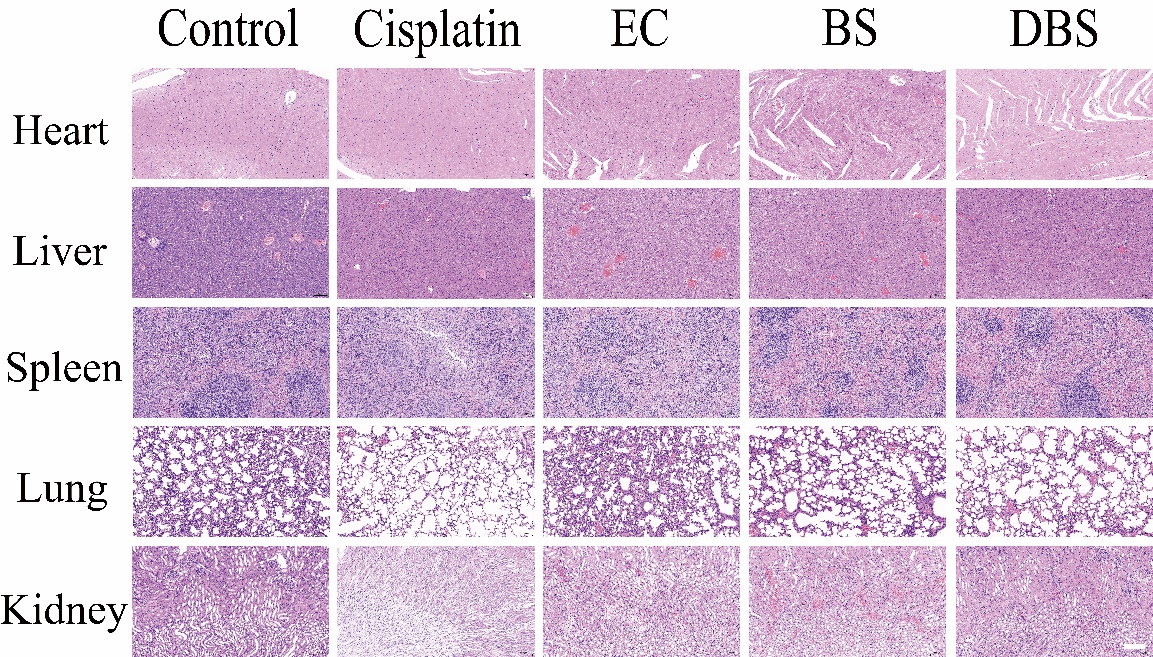


**Figure S32.** HE-stained sections of major organs after treatment completion with different drugs delivered via intratumoral injection.


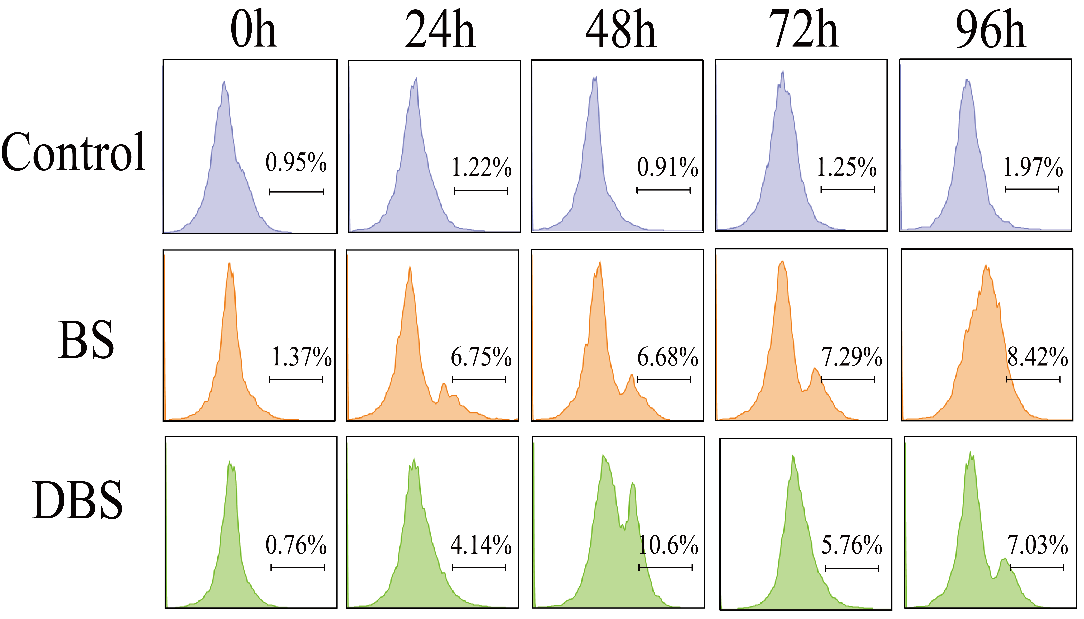


**Figure S33.** Fecal sample collection: Fecal samples were collected from each group at predefined time points post-treatment.These samples were cultured and analyzed using flow cytometry to quantify the BS or DBS fluorescence signal


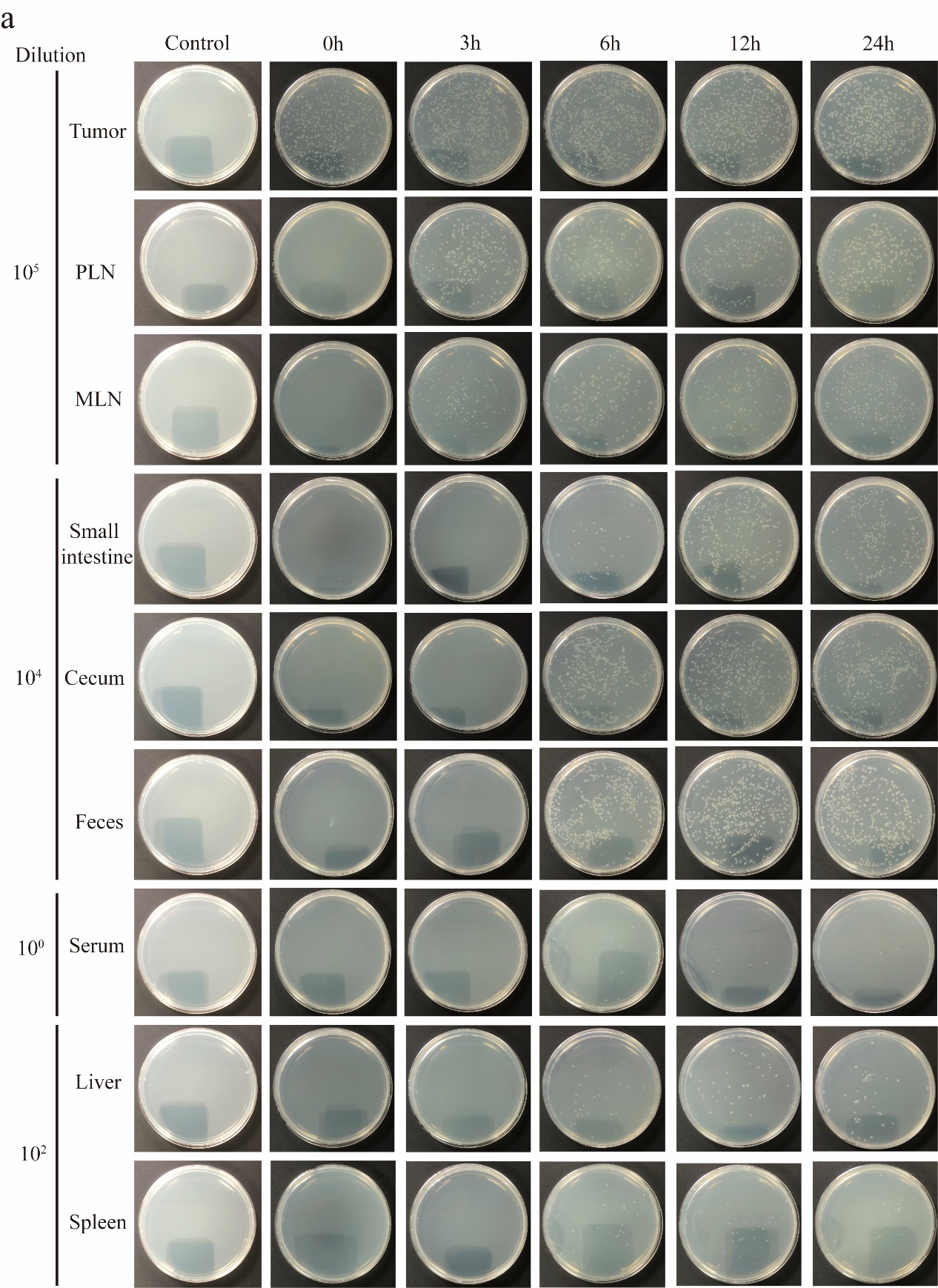


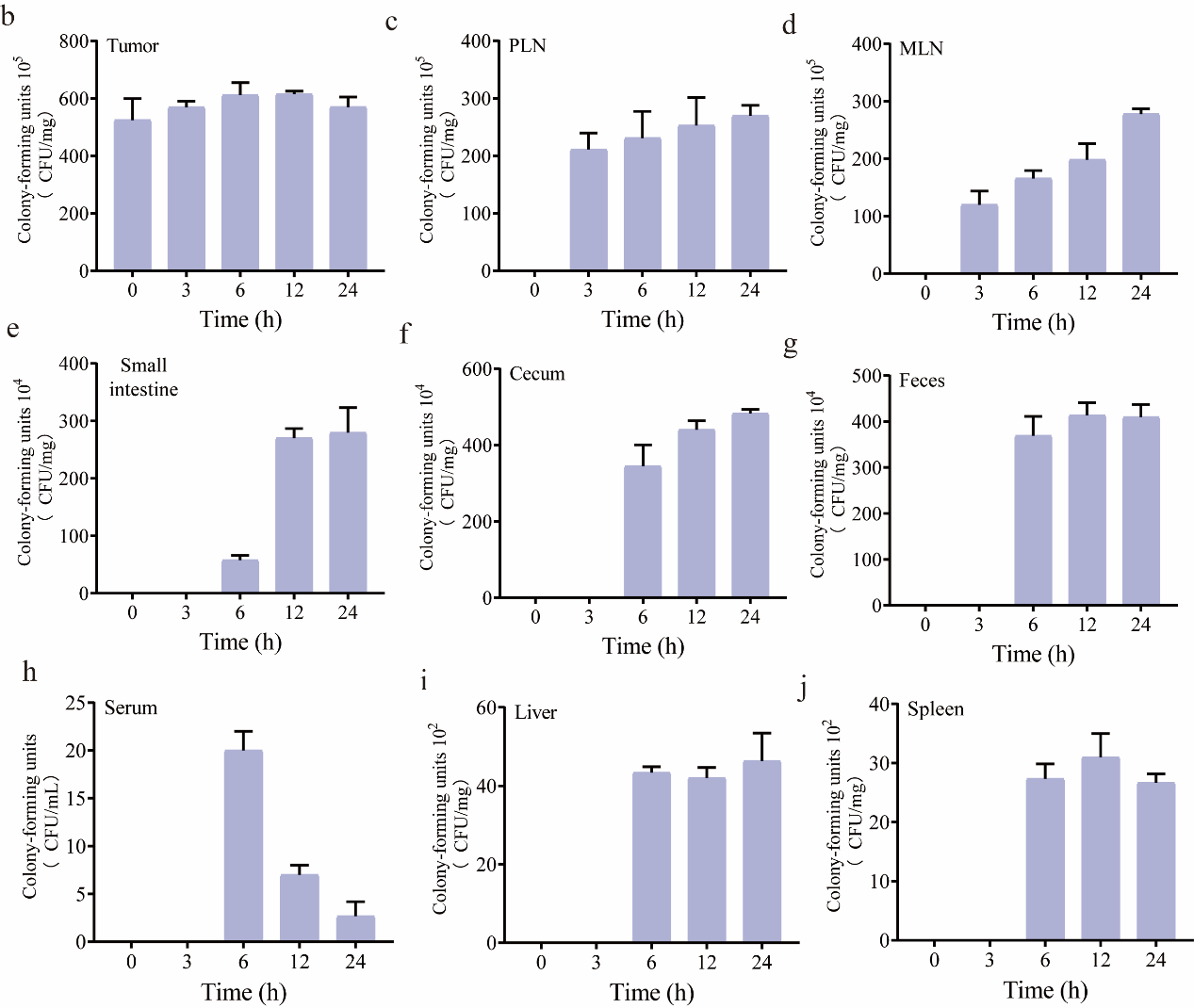


**Figure S34.** (a) Time‑course biodistribution of viable DBS‑GFP after intratumoral injection, assessed by colony‑forming‑unit (CFU) assays. (b–j) Quantitative CFU data (mean ± SD, n = 3 mice per time point) for each organ: (b) Tumour, (c) Popliteal lymph node (PLN), (d) Mesenteric lymph node (MLN), (e) Small‑intestine segment, (f) Cecum, (g) Feces, (h) Serum, (i) Liver, (j) Spleen.
